# Endocytic Adaptor Protein Tollip Inhibits Canonical Wnt Signaling

**DOI:** 10.1371/journal.pone.0130818

**Published:** 2015-06-25

**Authors:** Anna Toruń, Ewelina Szymańska, Irinka Castanon, Lidia Wolińska-Nizioł, Anna Bartosik, Kamil Jastrzębski, Magdalena Miętkowska, Marcos González-Gaitán, Marta Miaczynska

**Affiliations:** 1 International Institute of Molecular and Cell Biology, 02–109, Warsaw, Poland; 2 Department of Biochemistry, University of Geneva, 1211, Geneva, Switzerland; Comprehensive Pneumology Center, GERMANY

## Abstract

Many adaptor proteins involved in endocytic cargo transport exhibit additional functions in other cellular processes which may be either related to or independent from their trafficking roles. The endosomal adaptor protein Tollip is an example of such a multitasking regulator, as it participates in trafficking and endosomal sorting of receptors, but also in interleukin/Toll/NF-κB signaling, bacterial entry, autophagic clearance of protein aggregates and regulation of sumoylation. Here we describe another role of Tollip in intracellular signaling. By performing a targeted RNAi screen of soluble endocytic proteins for their additional functions in canonical Wnt signaling, we identified Tollip as a potential negative regulator of this pathway in human cells. Depletion of Tollip potentiates the activity of β-catenin/TCF-dependent transcriptional reporter, while its overproduction inhibits the reporter activity and expression of Wnt target genes. These effects are independent of dynamin-mediated endocytosis, but require the ubiquitin-binding CUE domain of Tollip. In Wnt-stimulated cells, Tollip counteracts the activation of β-catenin and its nuclear accumulation, without affecting its total levels. Additionally, under conditions of ligand-independent signaling, Tollip inhibits the pathway after the stage of β-catenin stabilization, as observed in human cancer cell lines, characterized by constitutive β-catenin activity. Finally, the regulation of Wnt signaling by Tollip occurs also during early embryonic development of zebrafish. In summary, our data identify a novel function of Tollip in regulating the canonical Wnt pathway which is evolutionarily conserved between fish and humans. Tollip-mediated inhibition of Wnt signaling may contribute not only to embryonic development, but also to carcinogenesis. Mechanistically, Tollip can potentially coordinate multiple cellular pathways of trafficking and signaling, possibly by exploiting its ability to interact with ubiquitin and the sumoylation machinery.

## Introduction

Adaptor proteins act as molecular scaffolds in various intracellular processes [1]. Usually lacking enzymatic activities, adaptors mediate protein-protein and protein-lipid interactions thanks to the presence of appropriate binding domains. As bridging molecules, adaptor proteins can integrate information and ensure cross-talk between different cellular pathways. On the other hand, they can also act independently in apparently unrelated processes. Such multiple, alternative functions of one protein in distinct subcellular compartments has been named “moonlighting” [2].

Endocytic adaptor proteins participate in all stages of endocytosis, including internalization of cargo and its subsequent intracellular sorting between endosomal and lysosomal compartments [3]. Some adaptor proteins can modulate the output of signal transduction cascades by trafficking specific signaling cargo, e.g. ligand-receptor complexes. However, several endocytic adaptors were described to exhibit alternative functions not linked to membrane transport, but related to cytoskeleton dynamics, nuclear signaling, transcription or mitosis [4–6].

Tollip (Toll-interacting protein) is an example of an endocytic adaptor protein. This ubiquitously expressed protein of 274 amino acids localizes to endosomes via interactions with Tom1, ubiquitin and clathrin [7, 8]. It contains an N-terminal Tom1-binding domain, a C2 domain interacting with phosphoinositides [9–11] and a C-terminal ubiquitin-binding CUE domain [12]. Tollip was originally identified as a negative regulator of the NF-κB pathway that binds interleukin-1 (Il-1) receptor I (Il-1RI) [13] and Toll-like receptors TLR2 and TLR4 [14]. At least some activities of Tollip in NF-κB signaling result from its function in endocytic trafficking of Il-1RI [15]. Similarly, Tollip modulates trafficking and degradation of transforming growth factor-β (TGF-β) receptor I (TβRI), and acts as an inhibitor of this pathway [14]. Another function of Tollip, promoting Rac1-dependent entry of bacteria into cells, depends on its trafficking function and endosomal interacting partners [16]. Recently, Tollip was reported to act as a ubiquitin-LC3 adaptor in autophagic clearance of cytotoxic polyQ proteins [17], thus potentially linking the autophagic and endocytic machineries.

In contrast, the proposed role of Tollip in control of sumoylation and nucleocytoplasmic shuttling of proteins is likely unrelated to its endocytic function. Tollip was shown to bind the components of the sumoylation machinery and to colocalize with SUMO-1 in the nuclear PML bodies [18]. Such a nuclear role of Tollip could exemplify its moonlighting. In general, a number of diverse interacting partners make Tollip a multifunctional adaptor acting in different cellular processes related or unrelated to its endocytic function [19]. At the level of an organism, a mouse knockout study demonstrated that Tollip is dispensable for embryonic development but regulates the magnitude and kinetics of cytokine production in response to Il-1β or lipopolysaccharide (LPS) [20], although a later discovery of multiple splicing isoforms of Tollip has put into question the completeness of the reported knockout [12].

Adaptor proteins perform multiple roles in intracellular signal transduction. Among such processes, Wnt signaling involves a complex network of pathways governing cell proliferation, differentiation and migration, as well as stem cell maintenance [21, 22]. In the canonical Wnt signaling, β-catenin is a key component which undergoes constant phosphorylation, ubiquitination and degradation in the absence of stimulation, while the pathway activation leads to its stabilization. This is initiated by Wnt ligands which bind to the Frizzled/LRP receptor complex and transmit signals via the Disheveled (Dvl) protein to destabilize the β-catenin degradation complex containing APC, Axin and GSK3β. Accumulated β-catenin translocates to the cell nucleus where it cooperates with TCF/LEF transcription factors to induce expression of target genes [23]. A large number of regulatory proteins participate in canonical Wnt signaling in addition to the generic core components. Wnt-dependent transcriptional programs are critical for embryonic development and organogenesis but also for tissue homeostasis and repair in an adult organism [24].

Here we report the results of a small-scale unbiased RNAi screen of endocytic adaptors which identified Tollip as a potential regulator of Wnt signaling. We further demonstrate that Tollip can function as an inhibitor of canonical Wnt signaling in cultured mammalian cells, both normal and cancerous, acting independently of dynamin-mediated endocytosis. We also reveal a role of Tollip in Wnt signaling in embryonic development of zebrafish: interfering with Tollip function in fish results in phenotypes reminiscent to those of canonical Wnt signaling mutants. Cumulatively, our results point to a previously unknown function of Tollip in intra- and intercellular signaling.

## Materials and Methods

### Ethics Statement

No specific ethics approval under Polish, Swiss and EU guidelines was required for this project, as all zebrafish used in this study were between 0 and 5 days old. Fish embryos were obtained in-house, as both the International Institute of Molecular and Cell Biology and the University of Geneva have institutional licenses to house and breed fish.

### Plasmids

Super8xTOPFlash and Super8xFOPFlash luciferase reporters and plasmids encoding Renilla (pRL-SV40), β-catenin S33Y (β-catenin S33Y-pCI-neo) and Wnt1 were a kind gift from Dr. Vladimir Korinek (Institute of Molecular Genetics, Prague); pCMV-Dvl2 from Prof. Jacek Otlewski (University of Wroclaw); plasmid encoding HA-tagged ubiquitin from Prof. Ivan Dikic (Goethe University Frankfurt); *ntl* plasmid from Dr. Carl-Philipp Heisenberg (Institute of Science and Technology, Austria). CCND1-Luc and AXIN2-Luc luciferase reporters were provided by Dr. Frank McCormick (Addgene plasmid 32727) [25] and Dr. Frank Costantini (Addgene plasmid 21275) [26], respectively. The following plasmids were previously described: *fgf8*-pCR2 [27], *goosecoid*-pBS [28], *cmlc2* [29] and dynamin2-K44A [30]. Full-length human Tollip was amplified from human cDNA by PCR using oligonucleotides: 5’-TACTGAGAATTCCCATGGCGACCACCGTCAG-3’ (forward, EcoRI site) and 5’-ATCTAGCGGCCGCCTTGTTGGGACAGCATTCCT-3’ (reverse, NotI site) for cloning into pcDNA3myc. Mutations in Tollip expression vector were introduced using the QuikChange Site-Directed Mutagenesis Kit (Stratagene). Tollip was mutated at M240A/F241A, R78A, R123A, H135A, K150E, R157A, K162A. Tollip deletion constructs were amplified from the plasmid expressing the full-length Tollip using the following oligonucleotides: for TOLLIPdel.1 5’-TACTGAGAATTCCCATGGCGACCACCGTCAG-3’ (forward), 5’-ATATAGCGGCCGCCTAACAGCGGGGCTGGGCGTTC-3’ (reverse), for TOLLIPdel.2 5’-TACTGAGAATTCCCATGGCGACCACCGTCAG-3’ (forward), 5’-ATATAGCGGCCGCCTACATGGCAGCTGGAAGCAGC-3’ (reverse), for TOLLIPdel.3 5’-GCGCTGAGAATTCATCGACTGAACATCACGGTGGTA-3’ (forward), 5’-ATCTAGCGGCCGCCTTGTTGGGACAGCATTCCT-3’ (reverse), for TOLLIPdel.4 5’-GCGCTGAGAATTCATCGACTGAACATCACGGTGGTA-3’ (forward), 5’-ATATAGCGGCCGCCTACATGGCAGCTGGAAGCAGC-3’ (reverse), for TOLLIPdel.5 5’-GAACTGAGAATTCATATGGTGATGCCACCCCAGC-3’ (forward), 5’-ATCTAGCGGCCGCCTTGTTGGGACAGCATTCCT-3’ (reverse) and cloned into EcoRI and NotI sites of pcDNA3myc. Full-length *z* *tollip* was amplified from zebrafish cDNA using the oligonucleotide pair: 5’-TACTGAGAATTCCCATGGCGACAACAATAAGCAC-3’ (forward), 5’-ATATACTCGAGTTTGGTATAAAGTCTGATGTGGAG-3’ (reverse) and cloned into EcoRI/XhoI sites of pCS+. All constructs were verified by sequencing. For mRNA synthesis, the plasmid carrying *ztollip* was linearized by NotI digest and transcribed by SP6 polymerase, while the plasmid carrying dynamin2-K44A was linearized by DraI and transcribed by T7 polymerase, using mMESSAGE mMACHINE Kit (Ambion) as previously described [31].

### Production of endoribonuclease-prepared siRNA (esiRNA)

esiRNA oligonucleotides were designed and prepared as described previously [32]. In brief, optimal esiRNA target regions with a length of 400–600 bp were selected using the DEQOR web server (http://deqor.mpi-cbg.de). T7 promoter sequence was added to the selected gene fragments by two PCR reactions. The first PCR reaction was carried out with the following gene-specific primer pairs tagged at 5’ end with a part of the T7 promoter (underlined): for Tollip 5’-TCACTATAGGGAGAGGACTGAACATCACGGTGGTAC-3’ (forward), 5’-TCACTATAGGGAGACGGCACCATGCCGGGGCTACAG-3 (reverse), for EGFP negative control 5’-GCTAATACGACTCACTATAGGGAGAGGTGAGCAAGGGCGAGGA-3’(forward), 5’-GCTAATACGACTCACTATAGGGAGACTACAGCTCGTCCATGCCGA-3’(reverse), for β-galactosidase #1 negative control 5’-TCACTATAGGGAGAGGCTGGCGTAATAGCGAAGAG-3’ (forward), 5’-TCACTATAGGGAGACCATTAAAGCGAGTGGCAACA-3’ (reverse), for β-galactosidase #2 negative control 5’-TCACTATAGGGAGAGGGTGAAGTGCCTCTGGATGT-3’ (forward), 5’-TCACTATAGGGAGACCCGCATCAGCAAGTGTATCT-3’ (reverse), for β-galactosidase #3 negative control 5’-TCACTATAGGGAGAGCACCCGAGTGTGATCATCTG-3’ (forward), 5’-TCACTATAGGGAGACCGGATAAACGGAACTGGAAA-3’ (reverse). In the second PCR reaction primers specific to T7 promoter were used: 5’-GCTAATACGACTCACTATAGGGAGAG-3’ (forward), 5’-GCTAATACGACTCACTATAGGGAGAC-3’ (reverse). Final PCR product served as a template in the *in vitro* transcription. The long dsRNA obtained was enzymatically digested with RNase III and purified. The concentration of esiRNA was determined by measuring OD260 nm.

### Small interfering RNA (siRNA)

Silencer Select siRNA oligonucleotides 5’-GCUGUUGAUUUAAGGCACAtt-3’ (S29037; designated #1) and 5’-GACUCUUUCUAUCUCGAGAtt-3’ (S29038; designated #2) against Tollip were purchased from Ambion. Two non-specific Silencer Select siRNA oligonucleotides (#4390843 and #4390846) served as negative controls.

### Cell culture

The following cell lines obtained from ATCC: DLD1 (#CCL-221), HCT116 (#CCL-247), and HEK293 (#CRL-1573) were maintained in Dulbecco’s modified Eagle’s medium (DMEM; Sigma-Aldrich) supplemented with 10% fetal bovine serum and 2 mM L-glutamine (Sigma-Aldrich). Wnt3a-expressing L cells (ATCC, #CRL-2648), and untransfected L cells (ATTC; #CRL-2647) were grown in DMEM supplemented additionally with 1 mM sodium pyruvate. Wnt3a-conditioned medium and control-conditioned medium was prepared according to the ATCC protocol.

### Cell transfection

Transient DNA transfections were done using Lipofectamine2000 (Life Technologies) or FuGene (Promega). SureFECT (SABiosciences) or Attractene Transfection Reagent (Qiagen) were used for co-transfection of DNA and siRNA. HiPerFect Transfection Reagent (Qiagen) was used for siRNA transfection. Transfections were performed according to manufacturers’ instructions and analyzed 24–48 h after DNA transfection or 72 h after siRNA transfection.

### Luciferase reporter assays

In overexpression experiments HEK293 cells were transfected with 200 ng of firefly luciferase reporter (Super8xTOPFlash, Super8xFOPFlash, CCND1-Luc or AXIN2-Luc), 50 ng of pRL-SV40 reporter vector and maximum 1 μg of the tested plasmid in a 24-well format (plasmids amounts were scaled down for experiments performed in a 96-well format). The amount of transfected DNA in all tested samples was kept constant by addition of an empty vector. In silencing experiments, RNAi at a final concentration of 33 nM were co-transfected with 50 ng of Super8xTOPFlash and 10 ng of pRL-SV40 reporter vectors in a 96-well format. In case of Wnt3a stimulation, cells were treated for the last 18 h before lysis, unless otherwise indicated. 24–72 hours post transfection cells were lysed in Passive Lysis Buffer (Promega) and either luciferase assay or Western blot analysis was performed. Luciferase reporter activity was measured in Firefly Buffer [50 mM Tris pH 7.8, 12 mM MgCl_2_, 10 mM DTT, 0.2 mM ATP (Sigma-Aldrich), 0.5 mM D-luciferin (Lux Biotechnology), 0.25 mM coenzyme A (Sigma-Aldrich)] or Renilla Buffer [50 mM Tris pH 7.8, 100 mM NaCl, 2.5 nM coelenterazine (Lux Biotechnology)]. The amount of light emitted in the reaction was determined in a microplate luminometer Centro XS3 LB960 (Berthold Technologies). The firefly luciferase activity derived from Super8xTOPFlash or Super8xFOPFlash reporter was normalized to its respective Renilla luciferase activity as a control for the transfection efficiency. Results are presented as the fold of untreated control in cells without stimulation and transfected with an empty vector or non-targeting RNAi, along with the luciferase reporter plasmids. If esiRNA was used, the pathway activity was normalized to the average of the four non-targeting controls (esiRNAs targeting EGFP and 3 different regions of β-galactosidase). All values are mean ± SEM from at least three independent experiments, each with three or four independent transfections performed in parallel.

### Setup of the esiRNA screen

esiRNAs targeting 80 selected human genes encoding endocytic proteins, esiRNAs against β-catenin, EGFP and 3 different regions of β-galactosidase were prepared as described above. HEK293 cells were transfected in a 96-well format with 33 nM esiRNA and reporter plasmids for 72 h, stimulated with Wnt3a-conditioned medium for the last 18 h prior to cell lysis and the luciferase assays were performed and analyzed as described above.

### Cell stimulation and inhibition of endocytosis

HEK293 cells were treated with Wnt3a-conditioned medium or control-conditioned medium for 1.5–18 h (depending on the assay) prior to cell lysis. LiCl was used at the final concentration of 50 mM for 5 h prior to cell lysis. Stimulation with Wnt1, Dvl2 and β-catenin S33Y was obtained by 24 h co-transfection of 25 ng of the respective plasmids in a 96-well format. To inhibit dynamin-dependent endocytosis cells were either transfected for 48 h with plasmid encoding the dominant-negative dynamin2-K44A mutant or treated with 30 μM dynasore (Merck) for 30 min and then for 5 h with Wnt3a-conditioned medium and dynasore.

### Quantitative PCR (qPCR)

Total RNA was isolated from HEK293 cells at 48 h of Tollip overexpression with the last 18 h of treatment with Wnt3a-conditioned medium, using GenElute Mammalian Total RNA Miniprep (Sigma-Aldrich). For cDNA synthesis random nonamers, oligo(dT)23 and M-MLV reverse transcriptase (Sigma-Aldrich) were used according to manufacturer’s instructions. To measure expression of Wnt target genes, the following pairs of oligonucleotides were used: *FGF9* 5’-ACCCGCAGTCACGGACTTGGA-3’ (forward), 5’-TGCTGACCAGGCCCACTGCTA-3’ (reverse), *NRP1* 5’-CCTGATGTTGGCCCTCACAT-3’ (forward), 5’-TGTCGGTGTAAAAAACCATGGA-3’ (reverse), *NKD1* 5’-AGAGCCCGGAAGGTGACA-3’ (forward), 5’- AGCGTGTCTCTCAACACGTC-3’ (reverse), *DKK1* 5’-CTCGGTTCTCAATTCCAACG-3’ (forward), 5’-GCACTCCTCGTCCTCTG-3’ (reverse) [33], *ACTB* 5’-CAGGTCATCACCATTGGCAAT-3’ (forward), 5’-TCTTTGCGGATGTCCACGT-3’ (reverse), *B2M* 5’- GGAGGCTATCCAGCGTACTC -3’ (forward), 5’- GAAACCCAGACACATAGCAATTC -3’ (reverse). The PCR reaction was performed with KAPA SYBR FAST qPCR kit (Kapa Biosystems) and 7900HT Fast Real-Time PCR thermocycler (Applied Biosystems) according to manufacturer’s recommendations. qPCR data were analyzed by the ΔΔCt method implemented in the Data Assist software (Applied Biosystems). The data were normalized according to the level of *ACTB* and *B2M*. All values are relative expression levels (fold change) compared to controls from cells incubated with control-conditioned medium and transfected with an empty plasmid (normalized to 1 for each gene). Results are presented as the mean fold change ± SEM from 3 independent experiments.

### Immunoblotting

Samples of 10–50 μg total protein were subjected to SDS-PAGE. Resolved proteins were transferred to nitrocellulose membrane (Whatman), which was blocked in 5% skim milk, probed with specific antibodies and detected by enhanced chemiluminescence on ImageQuant LAS 4000 (GE Healthcare Life Sciences) or Odyssey infrared imaging system (LI-COR Biosciences).

### Antibodies

The following primary antibodies were used: anti-Tollip (R&D Systems, #MAB4678; Santa Cruz, #sc-27315 and Sigma Aldrich, HPA038621), anti-ubiquitin (P4D1; Santa Cruz, #sc-8017), anti-ubiquitin Lys48-specific (Millipore; #05–1307), anti-ubiquitin Lys63-specific (Millipore; #05–1308), anti-myc (9E10, Millipore, #05–419), anti-HA (Santa Cruz, #sc-805), anti-β-catenin (BD Transduction Laboratories, #610154 and Santa Cruz, #sc-7199), anti-active β-catenin (Millipore, #05–665, clone 8E7), anti-dynamin (BD Transduction Laboratories, #610245), anti-α-tubulin (Sigma-Aldrich, #T5168), anti-β-actin (Sigma-Aldrich, #A5441), anti-GAPDH (Santa Cruz, #sc-25778) and anti-vinculin (Sigma-Aldrich, #V9131). All primary antibodies were used at dilution of 1:1000 for immunoblotting. Secondary peroxidase-conjugated antibodies were purchased from Jackson ImmunoResearch (#115-035-062 and 111-035-144) and fluorophore-conjugated from LI-COR Biosciences (#926-680-23 and #926–32212). All secondary antibodies for immunoblotting were used at dilution of 1:10000. For immunofluorescence, Alexa-conjugated secondary antibodies (Invitrogen; #A-21202, #A-31572) were used at dilution of 1:500.

### Immunoprecipitation

Cells were seeded on 100 mm culture plates and the next day transfected with plasmids encoding Tollip and HA-tagged ubiquitin (2.5 μg and 0.5 μg DNA, respectively). For immunoprecipitation of β-catenin cells were grown for 24 h and treated for the last 4 h before lysis with 5 μM MG132 (Enzo Life Sciences). For immunoprecipitation of Tollip, transfected cells were grown for 48 h and incubated for the last 18 h prior to lysis with Wnt3a-conditioned medium in the presence or absence of 5 μM MG132. After 24 or 48 h cells were washed once with PBS and lysed using RIPA buffer (1% Triton X-100, 0.5% sodium deoxycholate, 0.1% SDS, 50 mM Tris pH 8.0, 150 mM NaCl, 0.5 mM EDTA) containing 5 mM N-ethylmaleimide (Sigma-Aldrich) at 4°C. Lysates were clarified from cell debris by centrifugation and DNA was sheared using Qiashredder columns (Qiagen). After preclearance for 3 h with nonspecific immunoglobulins, bound proteins were depleted using Protein A or G agarose (Roche) for 1 h. Precleared cell lysates were then incubated overnight at 4°C with the indicated antibodies (anti-β-catenin from Santa Cruz sc-7199, anti-Tollip from Santa Cruz sc-27315) coupled to Protein A or G agarose. The agarose beads were washed four times using IP buffer (50 mM HEPES, 150 mM NaCl, 1 mM EDTA, 1 mM EGTA, 1% Triton X-100, 10% glycerol) before elution with 0.1 M glycine of pH 2.5. Finally samples were analyzed by SDS-PAGE and immunoblotting.

### Immunofluorescence and microscopy

HEK293 cells were seeded on 12-mm gelatin-coated coverslips in a 24-well plate (5x10^4^ cells/well for overexpression experiments; 10^4^ cells/well for silencing experiments). The coverslips were incubated in 0.2% gelatin (Sigma-Aldrich, G9391) for 30 min at 4°C prior to plating. The next day cells were transfected with DNA using Lipofectamine2000 or with siRNA using HiPerFect (Qiagen) according to the manufacturer’s instructions. After 24 h (overexpression) or after 72 h (silencing) cells were stimulated with Wnt3a-conditioned medium or control-conditioned medium for the indicated time, rinsed with PBS containing 1 mM CaCl_2_ and 0.5 mM MgCl_2_ and fixed with 3.6% paraformaldehyde (PFA) in PBS for 15 min at room temperature. Next, cells were permeabilized with 0.1% Triton X-100 and blocked using 10% fetal bovine serum in PBS for 30 min. Cells were further incubated overnight with primary antibodies against: β-catenin (BD Transduction Laboratories, #610154), Tollip (Sigma-Aldrich, HPA038621) or myc tag (Cell Signaling, #2278). Next day, cells were incubated with appropriate Alexa-conjugated secondary antibodies (30 min) and DAPI in 5% fetal bovine serum in PBS. For quantitative analysis at least ten randomly selected regions were imaged for each experimental condition. Single confocal plane images were acquired with Zeiss LSM710 microscope using 40x/1.30 oil immersion objective and 1024x1024 pixel resolution in ZEN 2009 software. Images were exported as TIFF and for visual presentation arranged in Adobe Photoshop CS4 Extended software with only linear adjustments. For quantification of β-catenin in the nucleus, 8-bit images were analyzed in ImageJ. Nuclei were identified as regions of interest based on DAPI staining. Fluorescence of β-catenin in the nuclei was measured and normalized to the nucleus area. Average β-catenin intensity in control cells plus standard deviation served as a cut-off for identification of β-catenin-positive nuclei. On average, over 200 nuclei per condition were analyzed in each experiment. The data are average percentage of β-catenin-positive nuclei or mean fluorescent intensity of nuclear β-catenin from 3 independent experiments ± SEM.

### Developmental staging and maintenance of zebrafish embryos

General maintenance, collection, and staging of the zebrafish were carried out according to the Zebrafish Book [34]. Wild-type (the AB strain) zebrafish embryos were maintained in Danieau zebrafish medium and grown at 28°C. The developmental stages were estimated based on time post-fertilization (hours or days; hpf or dpf) at 28°C as described [35].

### Immunohistochemistry (IHC)

Zebrafish embryos collected at different stages post fertilization were manually dechorionated, fixed with 4% paraformaldehyde and permeabilized in a graded series of methanol dilutions: 30%, 60%, 100% for 5 min each at RT and in 100% methanol for at least 1 h at -20°C. One dpf embryos were additionally treated with proteinase K at the concentration of 10 μg/ml for 5 min. Activity of endogenous peroxidases was inhibited by treatment with 0.3% H_2_O_2_ for 30 min. Embryos were blocked in 5% fetal bovine serum for 1 h and incubated with anti-Tollip antibody (R&D Systems, #MAB4678) diluted 1:250 overnight. Primary antibody was detected using Peroxidase Vectastain ABC Kit (Vector), according to the manufacturer’s recommendations. Secondary antibodies were detected by incubation in DAB (3,3'-diaminobenzidine) solution for 15 min and next in DAB solution plus 0.01% H_2_O_2_. The embryos were washed to stop the reaction and mounted in glycerol.

### Morpholino injections

Translation-blocking morpholino (MO) oligonucleotides complementary to 5’UTR and ATG regions of *ztollip* mRNA were obtained from Gene Tools: ATG1 5’-GTGCTTATTGTTGTCGCCATTCTGC-3’, UTR1 5’-TTATGATATGACGGACAGCGCCTGT-3’ (ATG1 and UTR1 target the first, longer transcript variant), ATG2 5’- CTGCTGCTGAGTCGGCATGATCCTC -3’ (targeting the second, shorter transcript variant). Dose-response experiments were carried out for all MOs to determine the highest nontoxic dose for use in further experiments. Up to 4 ng MO was microinjected into the yolk of 1-cell stage embryos (0.75 ng *ztollip* ATG1 MO, 4 ng *ztollip* UTR1 MO, 2 ng *ztollip* ATG2 MO). In double knock-down ISH experiments half of the MO amount was used. To determine the effects of gene silencing, a combination of the ATG2 and the UTR1 MO was used. MO-injected embryos were then manually dechorionated and either their phenotype was analyzed or they were subjected to ISH. To knock down Rab5 function, 1-cell stage embryos were injected with 4 ng of morpholino oligonucleotide directed against the coding region of the zebrafish *rab5a* (5'-GACAGTTGTCAATCACCCCGTCTTC-3'). The *β-catenin2* (*ctnnb2*) morpholino was previously described: 5’-CCTTTAGCCTGAGCGACTTCCAAAC-3’ [36].

### *In situ* hybridization (ISH)

*In situ* hybridization was carried out according to standard procedures [37]. For anti-sense *cmlc2*, *fgf8*, *goosecoid* and *ntl* RNA synthesis the appropriate plasmids were linearized and *in vitro* transcribed. Zebrafish embryos were fixed with 4% paraformaldehyde. One dpf embryos were additionally treated with proteinase K at the concentration of 10 μg/ml for 20 min. Prehybridization was performed at 65°C for 1 h. Embryos were incubated with digoxigenin-labeled RNA probes at 65°C overnight and blocked in 2% Blocking Reagent (Roche) for 5 h. Incubation with anti-digoxigenin alkaline phosphatase-conjugated antibodies (Roche #11093274910) diluted 1:5000 was performed at 4°C overnight. Antibodies were detected by incubation in the alkaline phosphatase substrate BM Purple (Roche). Embryos were washed to stop the reaction and mounted in glycerol.

### Statistical analysis

Statistical analyses were carried out using the software STATISTICA 8.0 (StatSoft). Comparisons among groups were made by nonparametric Mann-Whitney U test or Wilcoxon signed-rank test. Data are expressed as mean ± SEM from 3–4 independent experiments. Differences were considered statistically significant for *P*≤0.05.

## Results

### RNAi screen identifies Tollip as a negative regulator of canonical Wnt signaling

We set out to screen a subset of soluble endocytic proteins for a role in the regulation of canonical Wnt signaling, related to or independent of their function in endocytosis. The setup of a small-scale RNAi screen involved targeting 80 genes encoding adaptor and accessory proteins regulating endocytic internalization and cargo sorting. We employed a library of endoribonuclease-prepared short interfering RNAs (esiRNAs) to downregulate expression of the selected genes in HEK293 cells stimulated with Wnt3a. As readout, we used the Super8xTOPFlash luciferase reporter measuring the activity of TCF/LEF transcription factors [38]. The screen revealed a panel of genes whose knockdown decreased or increased the pathway activity (S1 Fig). Some of the hits, such as Tsg101 [39] and APPL1 previously reported by us [40], were known to positively regulate canonical Wnt signaling, arguing for the correct setup of the screen.

**Fig 1:**
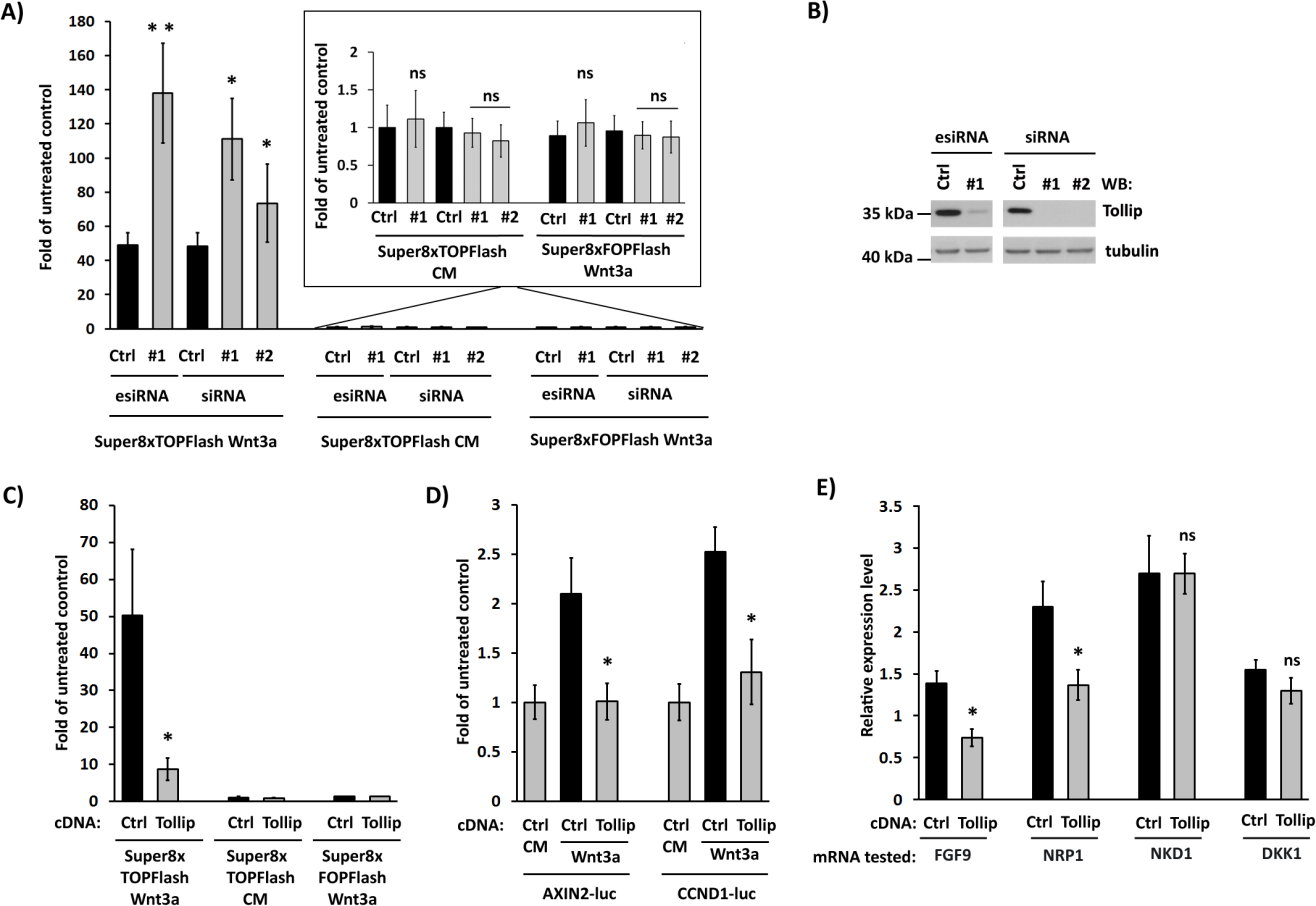
Tollip inhibits canonical Wnt signaling. (A) Depletion of Tollip potentiates the activity of Super8xTOPFlash luciferase reporter in HEK293 cells stimulated with Wnt3a-conditioned medium (left bars). Three RNAi reagents were used for Tollip silencing (esiRNA#1, siRNA #1 and #2) with appropriate non-targeting controls (Ctrl). No effects were observed in cells treated with control-conditioned medium (CM; middle bars and magnification above) or in Wnt3a-stimulated cells expressing mutated Super8xFOPFlash reporter (right bars and magnification above). The luciferase activity was measured as described in Materials and Methods. All values are expressed as fold of untreated control, i.e. cells incubated with control-conditioned medium and transfected with non-targeting RNAi reagents and the Super8xTOPFlash reporter. Data are mean ± SEM from 3 independent experiments; *P≤0.05, **P<0.01 (Wilcoxon signed-rank test). (B) Efficiency of Tollip silencing with RNAi reagents used in A was verified by Western blotting (WB), with α-tubulin serving as a loading control. (C) Overexpression of Tollip in Wnt3a-stimulated HEK293 cells inhibits the activity of Super8xTOPFlash reporter. Cells treated with control-conditioned medium (CM) or bearing mutated Super8xFOPFlash reporter served as controls, as in panel A. Ctrl, cells transfected with an empty pcDNA plasmid instead of Tollip-encoding construct. All values are expressed as fold of untreated control, i.e. cells incubated with control-conditioned medium and transfected with an empty plasmid and the Super8xTOPFlash reporter. (D) Overexpression of Tollip represses the activity of *AXIN2* and *CCND1* promoters (driving expression of luciferase) in HEK293 cells stimulated with Wnt3a. CM, cells treated with control medium. Ctrl, cells transfected with an empty pcDNA plasmid instead of Tollip-encoding construct. All values are expressed as fold of untreated control, i.e. cells incubated with control-conditioned medium and transfected with an empty plasmid and the respective luciferase reporter. (E) Overexpression of Tollip decreases mRNA levels of *FGF9* and *NRP1* genes (but not of *NKD1* or *DKK1*) in HEK293 cells stimulated with Wnt3a, measured by qPCR. Ctrl, cells transfected with an empty pcDNA plasmid instead of Tollip-encoding construct. All values are relative expression levels, compared to controls from cells incubated with control-conditioned medium and transfected with an empty plasmid (normalized to 1 for each gene; not shown). (C-E) Data are mean ± SEM from 3 independent experiments; *P≤0.05 (Mann-Whitney U test).

Among the potential inhibitors of the pathway (i.e. increasing the reporter activity upon knockdown), Tollip knockdown consistently increased the reporter activity with three independent RNAi reagents (an esiRNA pool and two single commercial siRNA oligonucleotides; Fig 1A and 1B). This effect was specific since Tollip silencing did not affect the control FOPFlash reporter bearing mutated TCF/LEF binding sites (Fig 1A, right bars). Furthermore, knockdown of Tollip did not increase basal Super8xTOPFlash reporter activity in HEK293 cells without Wnt3a stimulation (Fig 1A, middle bars). Conversely, overexpression of Tollip inhibited the reporter activity up to 5-fold in Wnt3a-treated cells, thus resulting in an opposite effect than silencing of Tollip expression (Fig 1C). Consistently, the observed inhibition was specific for the ligand-induced, but not basal pathway activity and for the wild-type, but not the mutated reporter (Fig 1C).

Overexpression of Tollip decreased the activity of promoters of two Wnt target genes: *AXIN2* [41] and *CCND1* (encoding cyclin D1) [25, 42] (Fig 1D). Similarly, the levels of expression of two other Wnt target genes, *FGF9* and *NRP1* [43–45], were reduced upon Tollip overproduction, as measured by qPCR (Fig 1E). However, expression of some other Wnt target genes, such as *NKD1* or *DKK1* [46], was not changed under these conditions, arguing that Tollip affects a specific subset of transcriptional targets of the Wnt pathway. Overall, gene expression analysis validated the results obtained in the Super8xTOPFlash reporter screen and allowed concluding that Tollip can exert an inhibitory activity on canonical Wnt signaling.

### The function of Tollip in Wnt signaling is independent of dynamin-mediated endocytosis

**Fig 2:**
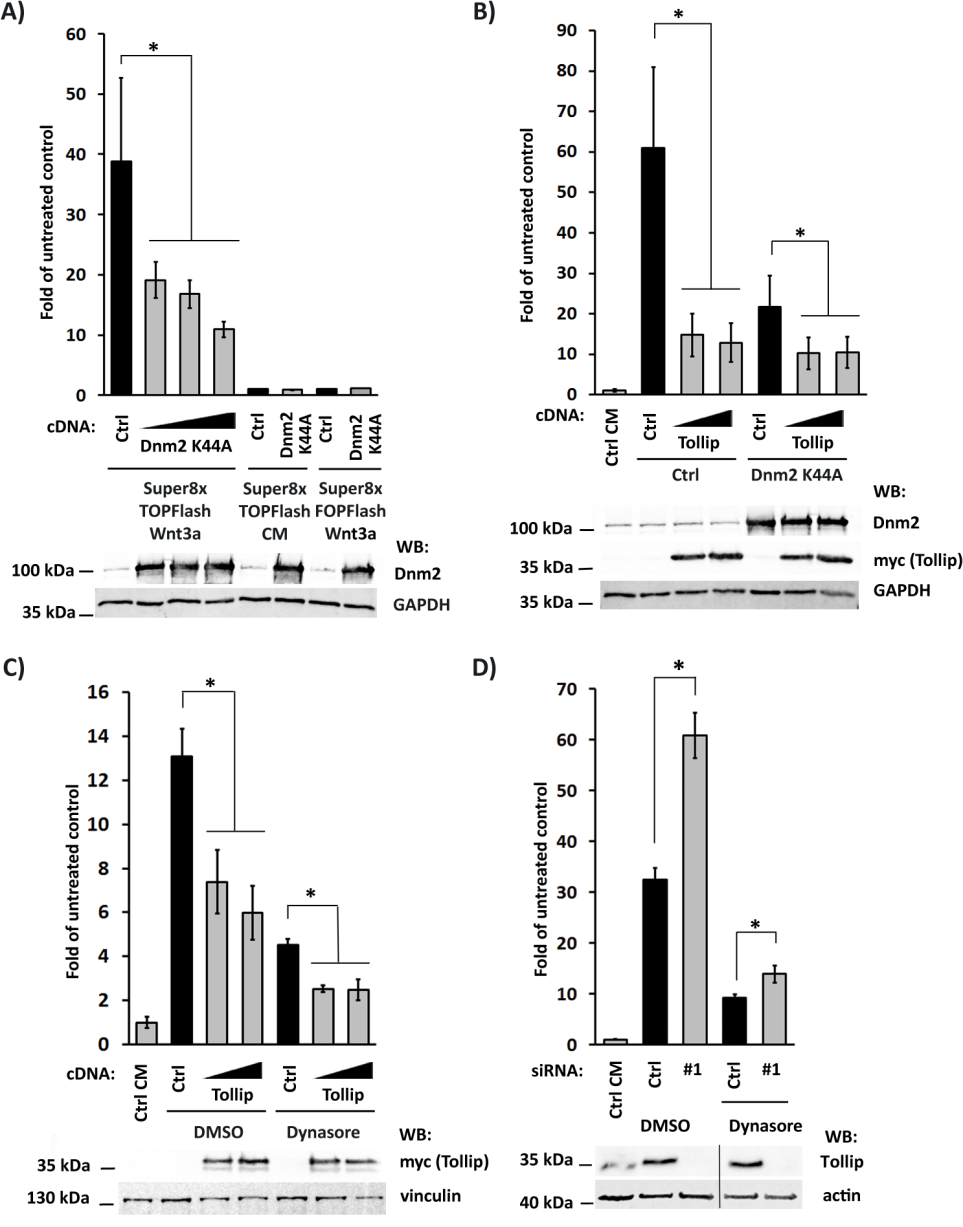
Tollip functions in Wnt signaling independently of dynamin-mediated endocytosis. (A) Expression of dynamin2-K44A mutant (Dnm2 K44A) inhibits the activity of Super8xTOPFlash luciferase reporter in a dose-dependent manner in HEK293 cells stimulated with Wnt3a-conditioned medium. No effects were observed in cells treated with control-conditioned medium (CM) or in Wnt3a-stimulated cells expressing mutated Super8xFOPFlash reporter. All values are expressed as fold of untreated control, i.e. cells incubated with control-conditioned medium and transfected with an empty plasmid and the Super8xTOPFlash reporter. Protein levels of overexpressed Dnm2-K44A were verified by Western blotting, with GAPDH as a loading control (lower panel). (B) Overexpression of Tollip further represses the Super8xTOPFlash reporter activity in Wnt3a-stimulated cells HEK293 cells overproducing Dnm2-K44A. Ctrl, cells transfected with an empty pcDNA plasmid instead of Tollip-encoding construct. All values are expressed as fold of untreated control, i.e. cells incubated with control-conditioned medium (CM) and transfected with an empty plasmid and the Super8xTOPFlash reporter. Protein levels of overexpressed Dnm2-K44A and Tollip (detected via its myc tag) were verified by Western blotting, with GAPDH as a loading control (lower panel). (C) Overexpression of Tollip further represses the Super8xTOPFlash reporter activity in HEK293 cells stimulated with Wnt3a for 5 h and concomitantly treated with dynasore or DMSO as vehicle control. Ctrl, cells transfected with an empty pcDNA plasmid instead of Tollip-encoding construct. All values are expressed as fold of untreated control, i.e. cells incubated with control-conditioned medium (CM) and DMSO, and transfected with the Super8xTOPFlash reporter. Protein levels of overexpressed Tollip (detected via its myc tag) were verified by Western blotting, with vinculin as a loading control (lower panel). (D) The activity of Super8xTOPFlash luciferase reporter in HEK293 cells depleted of Tollip (by siRNA #1), stimulated with Wnt3a and treated with dynasore for 5 h. Ctrl, cells transfected with non-targeting siRNA. All values are expressed as fold of untreated control, i.e. cells incubated with control-conditioned medium (CM) and DMSO, and transfected with the Super8xTOPFlash reporter. Knockdown efficiency of Tollip was verified by Western blotting, with actin as a loading control (lower panel). (A-D) Data are mean ± SEM from 3 independent experiments; *P≤0.05 (Mann-Whitney U test or Wilcoxon signed-rank test in B).

We then verified whether the function of Tollip in Wnt signaling is due to its activity in endocytic processes. Endocytosis was shown to regulate the canonical Wnt signaling, although its role seems to be either stimulatory or inhibitory depending on the cell type [22, 47–49]. In Wnt3a-stimulated HEK293 cells, expression of dominant-negative K44A mutant of dynamin2 [50] reduced the reporter activity (Fig 2A), without affecting the levels of Tollip protein (S2A Fig). This effect was independently confirmed by treating cells with a pharmacological inhibitor of dynamin, dynasore [51], which also efficiently blocked the reporter stimulation (Fig 2C). Inhibition of endocytosis occurred in both cases (overexpression of dynamin mutant and dynasore treatment), as internalization of transferrin was strongly reduced (data not shown). However, when Tollip was overexpressed together with the dynamin-K44A mutant or along with the dynasore application, its negative effects on the TCF/LEF reporter were still clearly visible (Fig 2B and 2C). Conversely, Tollip depletion could potentiate the reporter activity in dynasore-treated cells (Fig 2D). These data indicate that the inhibitory activity of Tollip in canonical Wnt signaling is independent of dynamin-mediated endocytosis, thus likely representing a moonlighting function of this protein.

### Ubiquitin binding is important for the function of Tollip in Wnt signaling

To address possible mechanisms underlying the moonlighting action of Tollip, we first studied which of its domains (engaged in well-known interactions with other molecules and pathways) might be important for Wnt signaling. Of the three domains of Tollip, the N-terminus is responsible for association with TOM1 protein, the middle C2 domain binds phosphoinositides, while the C-terminal CUE domain interacts with ubiquitin [7, 11].

**Fig 3:**
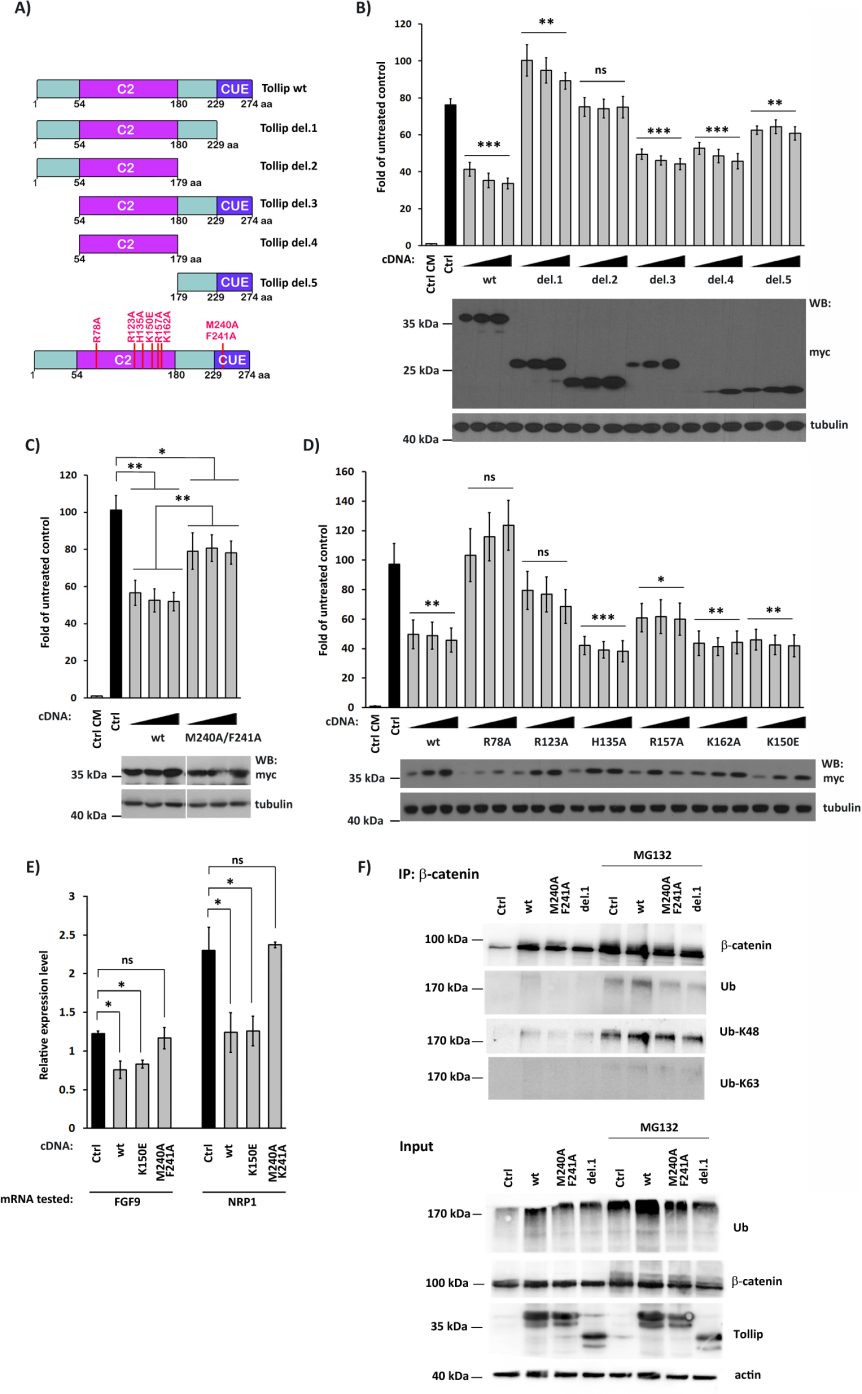
Tollip requires an intact ubiquitin-binding domain to function in canonical Wnt signaling. (A) Schematic representation of deletion and point mutants of human Tollip. (B-D) myc-tagged Tollip wild-type (wt) and mutants were tested in the Super8xTOPFlash reporter assay (upper panels; B, deletion mutants; C, ubiquitin-binding deficient M240A/F241A mutant; D, phosphoinositide-binding deficient point mutants). Increasing amounts of mutant-encoding plasmids were transfected. Ctrl, cells transfected with an empty pcDNA plasmid instead of a Tollip-encoding construct. All values are expressed as fold of untreated control, i.e. cells incubated with control-conditioned medium (CM) and transfected with an empty plasmid and the Super8xTOPFlash reporter. Data are mean ± SEM from 3 (C) or 4 (B, D) independent experiments; *P≤0.05, **P<0.01, ***P<0.001 (Mann-Whitney U test). Expression of mutated Tollip proteins was verified by Western blotting (WB) using anti-myc antibodies, with α-tubulin as a loading control (lower panels). (E) Expression of *FGF9* and *NRP1* genes upon overexpression of Tollip wt, K150E and M240A/F241A mutants in Wnt3a-stimulated HEK293 cells (Ctrl), measured by qPCR. All values are relative expression levels, compared to controls from cells incubated with control-conditioned medium and transfected with an empty plasmid (normalized to 1 for each gene; not shown). Data are mean ± SEM from 3 independent experiments; *P≤0.05 (Mann-Whitney U test). (F) Immunoprecipitation of β-catenin from lysates of HEK293 cells transfected with an empty plasmid (Ctrl) or with constructs expressing wild-type Tollip (wt), M240A/F241A mutant or deletion 1 (del.1) mutant. Cells were either untreated or incubated with MG132 for 4 h before lysis. Upper panel; immunoprecipitates were probed with antibodies against β-catenin, total ubiquitin (Ub), its K48-linked (Ub-K48) or K63-linked (Ub-K63) chains. Lower panel; 10% of starting lysates taken for immunoprecipitation (input) were blotted against total ubiquitin (Ub), β-catenin and Tollip, with actin as a loading control.

Deletion mutagenesis was performed and the resulting five mutants (Fig 3A) were tested for their effects in the reporter assay (Fig 3B). The mutants devoid of the CUE domain (del.1 and del.2) lost the ability to inhibit the Wnt pathway activity. Instead, inhibition was retained in a mutant lacking the N-terminal TOM1-binding motif (del.3). Interestingly, the C2 domain alone (del.4) also inhibited the reporter activity, in contrast to a minor effect of the C-terminal fragment containing the CUE domain (del.5). We therefore concluded that the interaction with TOM1 via the N-terminus of Tollip is not important for the regulation of Wnt signaling. Instead, both the C2 and the CUE domains mediate this function of Tollip, suggesting that binding of phosphoinositides and/or ubiquitin may be involved. Consistent with this idea, both types of molecules are implicated in Wnt signaling. Phosphatidylinositol (4,5)-bisphosphate (PI4,5P_2_) is produced at the plasma membrane upon Wnt3a stimulation to drive formation of LRP6 signalosomes [52–54], while ubiquitin plays a number of roles, either proteolytic or regulatory, at various stages of the canonical Wnt cascade [55].

We confirmed these results with a panel of previously characterized point mutations of Tollip (Fig 3A). A double mutant M240A/F241A within the CUE domain perturbs an association with ubiquitin [15]. Six individual point mutations within the C2 domain (R78A, R123A, H135A, K150E, R157A, K162A) inhibit binding of Tollip to phosphatidylinositol 3-phosphate (PI3P) and two of them (R78A, R123A) additionally destabilize an interaction with PI4,5P_2_ [9, 10]. When tested in the reporter assays, the ubiquitin binding-deficient M240A/F241A mutant did not inhibit the pathway activity as efficiently as the wild-type protein (Fig 3C). This confirms the results of deletion mutagenesis and indicates that ubiquitin binding is important for the repressive function of Tollip in Wnt signaling. Of note, we detected K48- and K63-linked ubiquitin chains in immunoprecipitates of Tollip (S2B Fig) which indicates its association with ubiquitinated proteins, as reported [17], and/or a direct modification of Tollip itself.

Of the six phosphoinositide-binding mutants, two (R78A, R123A) were unable to inhibit the reporter activity, although the levels of R78A overexpression were the lowest (Fig 3D). The other mutants (expressed at levels comparable to each other and to the wild-type form) exhibited either the wild-type (H135A, K162A, K150E) or slightly lower (R157A) activity. These results indicate that, if at all, association with PI4,5P_2_ may be of some importance for the function of Tollip in Wnt signaling, consistent with the role of this lipid in Wnt signaling [52–54]. However, binding of PI3P appears not to be required for the Tollip function in Wnt inhibition. Considering that PI3P is predominantly enriched in endosomal membranes [56], this would further support our conclusion that the endocytic and Wnt regulatory functions of Tollip are independent of each other. Consistent with the reporter data, when expression of Tollip target genes *FGF9* and *NRP1* was tested, the ubiquitin-binding M240A/F241A mutant failed to inhibit expression of the two targets, in contrast to the wild-type and the K150E mutant (Fig 3E). Finally, increased K48-linked ubiquitination of β-catenin and/or its associated proteins was detected in β-catenin immunoprecipitates upon overexpression of the wild-type Tollip but not of its mutants deficient in ubiquitin binding (M240A/F241A and del.1 devoid of the CUE domain; Fig 3F). These data are in agreement with an inhibitory activity of Tollip on Wnt signaling because enhanced polyubiquitination of β-catenin favors its degradation, thus restraining the pathway. Cumulatively, these data show that the ability of Tollip to bind ubiquitin is required for its function in Wnt signaling.

### Tollip regulates a pool of active β-catenin in Wnt-stimulated cells

**Fig 4:**
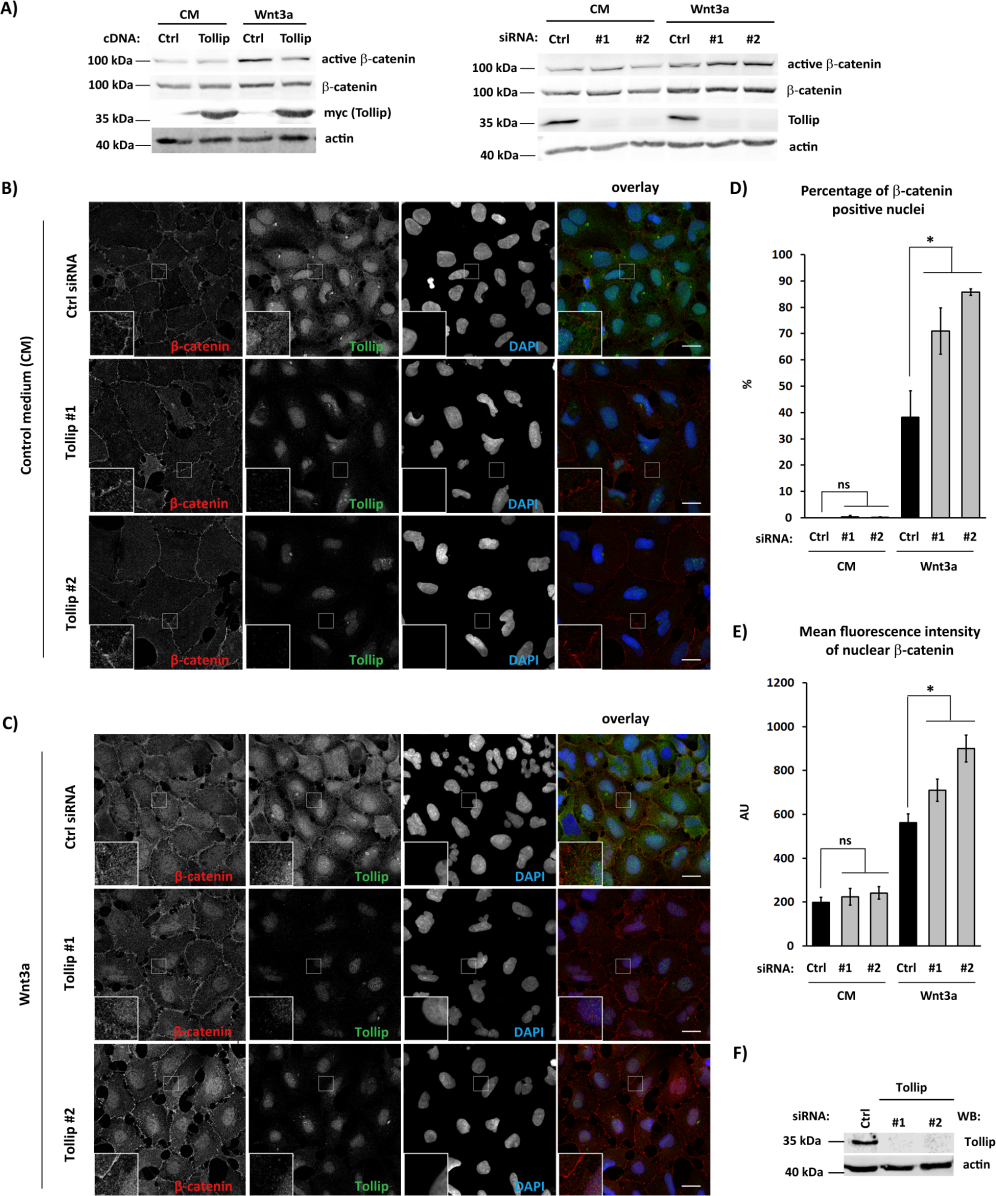
Tollip affects a pool of active β-catenin. (A) Levels of active and total β-catenin were investigated in HEK293 cells treated with Wnt3a- or control conditioned (CM) medium for 1.5 h upon overexpression of myc-Tollip (left panel) or siRNA-mediated depletion of Tollip (right panel). Immunoblotting was performed with the indicated antibodies, with actin as a loading control. (B-C) HEK293 cells were transfected with siRNA silencing Tollip (siRNA #1 or #2) or with appropriate non-targeting control (Ctrl). After 72 h they were stimulated with control medium (CM; B) or Wnt3a-conditioned medium (C) for 10 h. Cells were immunostained for β-catenin and Tollip, with DAPI indicating cell nuclei. Residual nuclear staining visible in Tollip-depleted cells represents an unspecific signal of the Tollip antibody (compare silencing efficiency visualized by immunoblotting in F). Scale bar 20 μm. (D-E) Acquired images were analyzed with ImageJ to calculate the percentage of β-catenin-positive nuclei (D) or mean fluorescence intensity of nuclear β-catenin (expressed in arbitrary units, AU; shown in E), as described in Materials and Methods. Data are mean ± SEM from 3 independent experiments; *P≤0.05 (Mann-Whitney U test). (F) Efficiency of Tollip silencing with RNAi reagents used in (A) was verified by Western blotting (WB), with actin serving as a loading control.

We wished to identify components of the Wnt transduction pathway affected by Tollip. First, we assessed the impact of Tollip on the level of β-catenin, both total and its active (non-phosphorylated on Ser37/Thr41) form. In Wnt3a-treated cells, overexpression of Tollip reduced the pool of active β-catenin without altering its total levels (Fig 4A, left panel). Conversely, depletion of Tollip increased amounts of active β-catenin but not its overall pool (Fig 4A, right panel). These observations are consistent with the role of Tollip as a negative regulator of the pathway.

As activation of β-catenin leads to its translocation to the nucleus, we verified whether Tollip influenced this process, employing quantitative immunofluorescence microscopy. As expected, silencing of Tollip expression potentiated accumulation of β-catenin in nuclei of Wnt3a-treated cells (Fig 4C–4F). This was manifested by increased percentage of β-catenin-positive nuclei (Fig 4D) and higher mean fluorescence intensity of nuclear β-catenin (Fig 4E). No such changes were observed in cells without Wnt stimulation (Fig 4B, 4D and 4E), consistent with the notion that Tollip affects the ligand-induced, but not basal pathway activity (Fig 1A and 1C). We could not, however, measure unequivocal effects of Tollip overexpression on β-catenin nuclear localization in Wnt3a-treated cells (S3 Fig), possibly due to variable levels of protein overproduction between individual cells upon transient transfection. Cumulatively, the biochemical and microscopical data suggest that in Wnt3a-treated cells Tollip can counteract the activation of β-catenin and its subsequent nuclear translocation, thus inhibiting the pathway.

### Tollip can also inhibit ligand-independent, constitutive Wnt signaling

**Fig 5:**
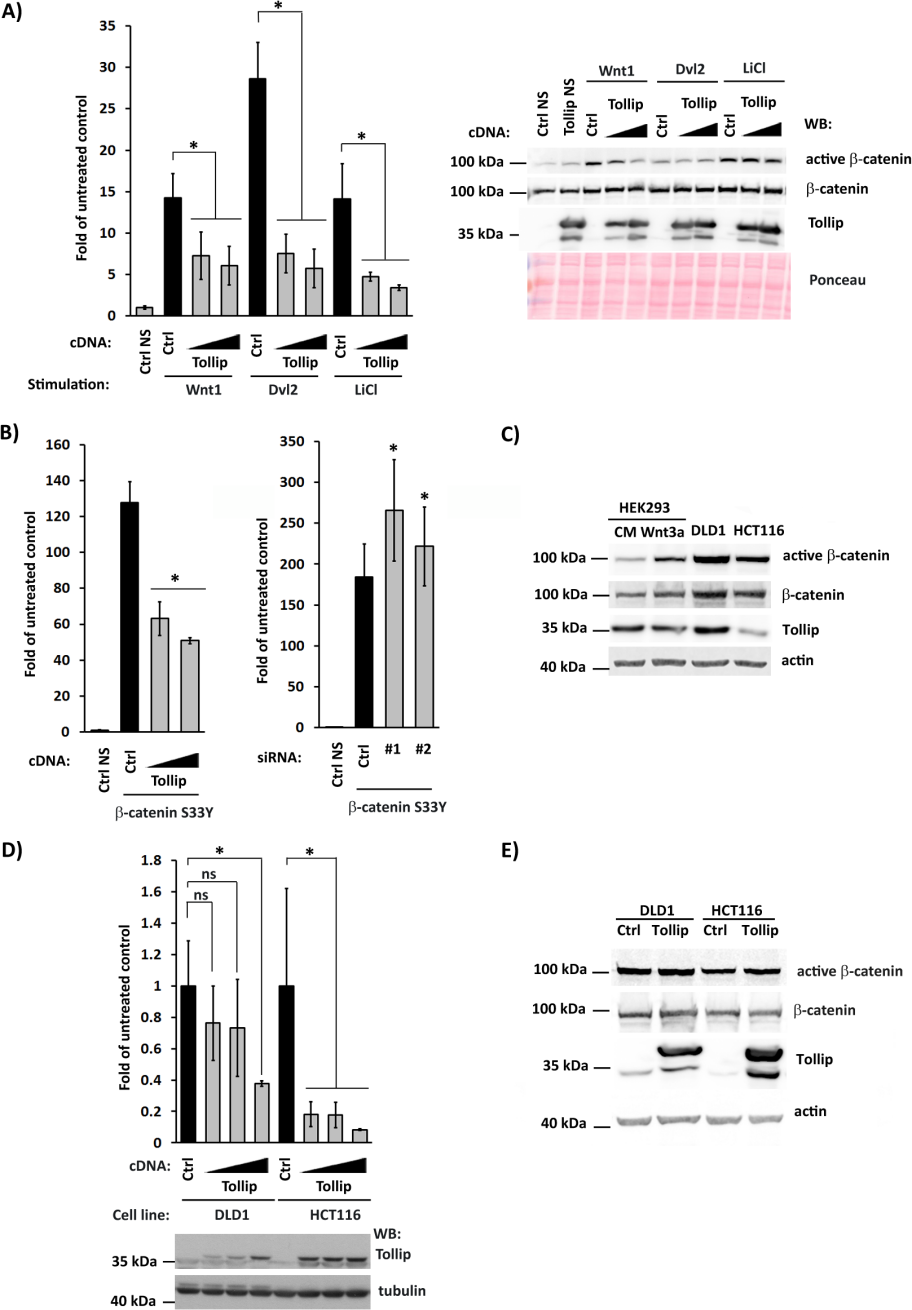
Tollip inhibits ligand-independent Wnt signaling in HEK293 and cancer cell lines. (A) Overexpression of Tollip inhibits the activity of Super8xTOPFlash luciferase reporter in HEK293 cells stimulated by overexpression of Wnt1 or Disheveled 2 (Dvl2), or LiCl treatment (left panel). Two amounts of Tollip-encoding plasmids were tested. All values are expressed as fold of untreated control, i.e. the reporter activity in cells without any stimulant which was normalized to 1 (Ctrl NS). Ctrl denotes the reporter activity in cells treated with a given stimulant but transfected with an empty plasmid instead of Tollip. Data are mean ± SEM from 3 independent experiments; *P≤0.05 (Wilcoxon signed-rank test). Right panel: Levels of active and total β-catenin were investigated in lysates of cells tested in the reported assay. NS, non-stimulated cells. Immunoblotting was performed with the indicated antibodies, with Ponceau staining shown as a loading control. (B) Tollip inhibits Wnt signaling in HEK293 cells stimulated by overexpression of β-catenin-S33Y mutant. Overexpression of Tollip (left panel) or its siRNA-mediated silencing (right panel) alters the activity of Super8xTOPFlash luciferase reporter. All values are expressed as fold of untreated control, i.e. the reporter activity in cells without β-catenin-S33Y overexpression which was normalized to 1 (Ctrl NS). Ctrl denotes the reporter activity in cells treated with β-catenin-S33Y overexpression and transfected with an empty plasmid instead of Tollip (left panel) or non-targeting siRNA control (right panel). Data are mean ± SEM from 3 independent experiments; *P≤0.05 (Wilcoxon signed-rank test). (C) Levels of active and total β-catenin, and of Tollip were investigated in HEK293 cells treated with Wnt3a- or control conditioned (CM) medium for 6 h, and in unstimulated DLD1 and HCT-116 cells. Immunoblotting was performed with the indicated antibodies, with actin as a loading control. (D) Overexpression of Tollip suppresses the activity of Super8xTOPFlash luciferase reporter in cancer cell lines DLD1 and HCT116 without exogenous stimulation (upper panel). Increasing amounts of Tollip-encoding plasmid were tested. The reporter activity in cells transfected with an empty plasmid was normalized to 1 for each line (Ctrl). Data are mean ± SEM from 3 independent experiments; *P≤0.05 (Mann-Whitney U test). Lower panel: the levels of Tollip overexpression were verified by Western blotting (WB), with α-tubulin serving as a loading control. (E) Levels of active and total β-catenin, and of Tollip were investigated in DLD1 and HCT-116 cells overexpressing Tollip or transfected with an empty plasmid (Ctrl). Immunoblotting was performed with the indicated antibodies, with actin as a loading control.

The Wnt pathway can also be induced in the absence of extracellular ligands by alterations in intracellular signaling components. We thus verified whether Tollip can also inhibit ligand-independent Wnt signaling. First, we employed also two intracellular stimuli such as overexpression of a pathway activator Disheveled 2 (Dvl2) [57] and treatment of cells with LiCl which inhibits GSK3β [58, 59], in comparison to another canonical ligand (Wnt1). In all three cases, overexpression of Tollip was still able to inhibit the TCF/LEF reporter activity (Fig 5A), as initially observed for cells stimulated with Wnt3a (Fig 1C). In case of Wnt1, this inhibition was also accompanied by a visible decrease in the levels of active β-catenin without changes in its total amounts (Fig 5A), similarly to effects exerted by Wnt3a (Fig 4A). These data argue that upon ligand stimulation, Tollip counteracts the activation of β-catenin without affecting its stabilization. However, no changes in β-catenin status were apparent in lysates of cells overexpressing Dvl2 or treated with LiCl (Fig 5A), suggesting that Tollip can exert its inhibitory action at yet another step in the pathway, occurring after β-catenin stabilization.

Ligand-independent stabilization of β-catenin leads to permanent activation of the Wnt pathway and is a common pathological mechanism of numerous diseases, including cancer [23, 24]. To test whether Tollip could inhibit constitutive Wnt signaling, we first overproduced a degradation-resistant β-catenin mutant S33Y [60] in HEK293 cells. Under these conditions, overexpression of Tollip inhibited, while its silencing potentiated the TCF/LEF reporter activity (Fig 5B). These effects argued that Tollip can interfere with ligand-independent Wnt signaling.

This conclusion was further supported by testing colorectal cancer cell lines in which canonical Wnt signaling is constitutively active due to mutations either in β-catenin (HCT116 line) or in APC (DLD1 line) [60, 61]. Indeed, the levels of total and active β-catenin were increased in these lines, compared to Wnt3a-treated HEK293 cells (Fig 5C). In both lines, overexpression of Tollip inhibited the TCF/LEF reporter activity, with the strongest effects observed in better transfectable HCT116 cells (Fig 5D). However, overproduced Tollip did not change the levels of total or active β-catenin (Fig 5E), arguing that it inhibits the pathway at a step downstream of β-catenin stabilization.

Overall, our results suggest that an inhibitory action of Tollip in Wnt signaling can be exerted at two steps of the pathway. In ligand-stimulated cells, Tollip lowers the pool of active (non-phosphorylated) β-catenin without altering its total levels. Interestingly, in macrophages Tollip was reported to participate in the activation of GSK3β [62]. GSK3β inhibits Wnt signaling by promoting phosphorylation and degradation of β-catenin [23]. Therefore, Tollip could potentially impair activation of β-catenin via increased GSK3β activity. In parallel, Tollip can also inhibit the Wnt pathway acting downstream of β-catenin stabilization, as observed under conditions of ligand-independent, constitutive Wnt signaling. This could possibly involve regulation of nuclear activities of β-catenin and its associated proteins by Tollip which can also localize to the cell nucleus [18].

In general, our data support a widespread role of Tollip-mediated regulation of Wnt signaling, both ligand-induced and ligand-independent, in different cell types, including cancer cells. Consistently, decreased expression of Tollip was reported during oncogenic progression from normal colon mucosa to adenomas and carcinomas [63]. Activation of canonical Wnt signaling is a well-documented pathological mechanism in colon cancer [64] and lower levels of an inhibitory protein such as Tollip could additionally contribute to this process.

### The function of Tollip in embryonic development of zebrafish

In the adult, Wnt signaling drives gene expression to maintain stem cell homeostasis and when aberrant leads to carcinogenesis. However, the Wnt pathway controls also body patterning during early vertebrate development [65]. To address whether Tollip-dependent regulation of Wnt signaling is evolutionarily conserved, we analyzed the developmental role of Tollip in zebrafish embryos. In this organism, the canonical Wnt signaling pathway is essential for the specification of ventral and posterior fates (for review see [66]). Indeed, inhibition of the canonical Wnt signaling by either Wnt3/Wnt8 downregulation or missexpression of the Wnt signaling inhibitor Dickkopf1 (Dkk1) consistently reduces the formation of the posterior body [67–69].

**Fig 6:**
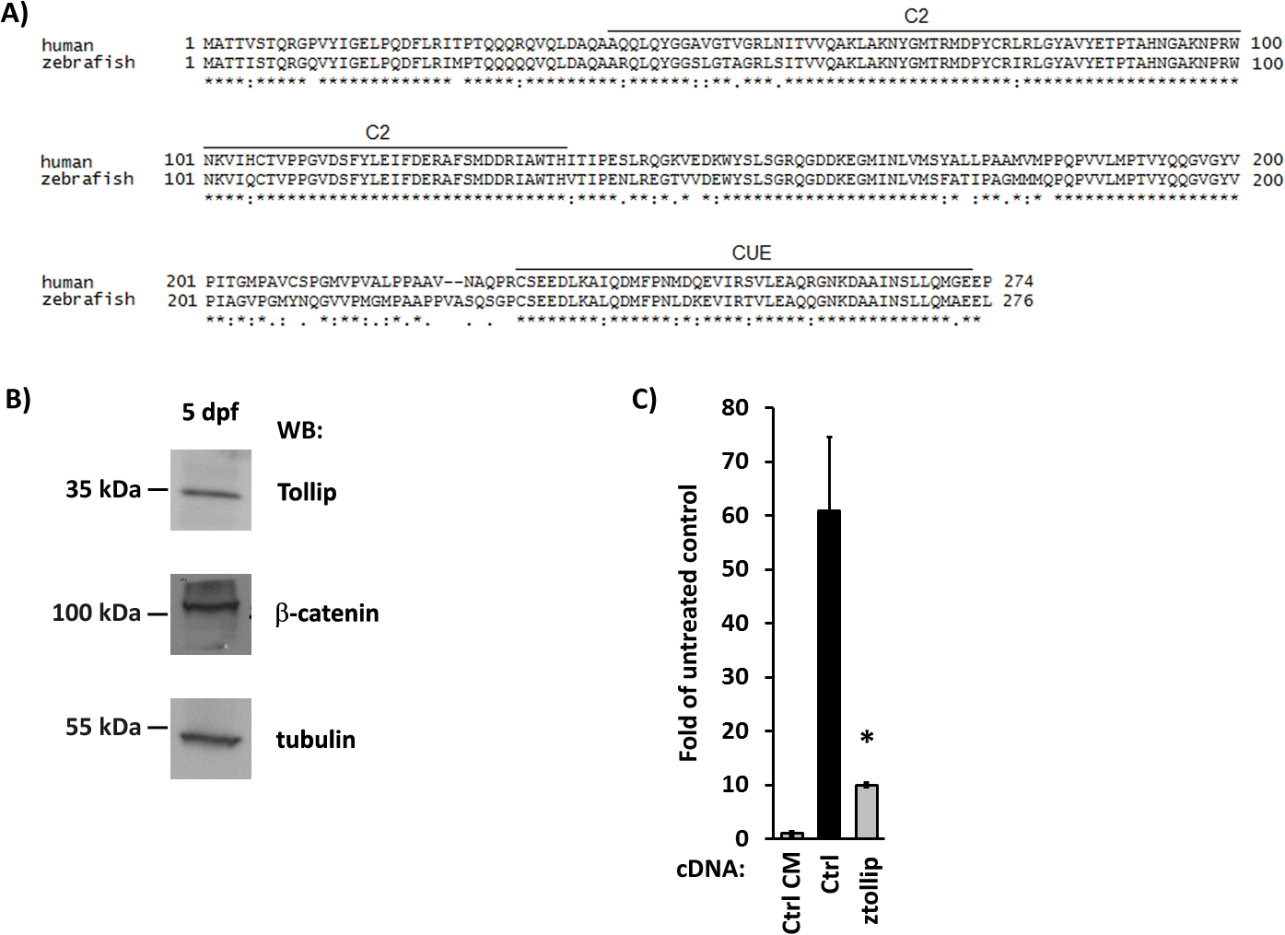
Zebrafish Tollip (zTollip) shows conservation of both sequence and function with the human ortholog. (A) Protein sequence alignment between human Tollip (NP_061882.2) and zebrafish Tollip (NP_996944.1) was done using a T-Coffee web server [88]. Identical (asterisk), conserved (double dot), and partially conserved (single dot) residues are indicated below amino acids symbols. The lines above mark the highly conserved C2 and CUE domains. (B) Antibodies against human Tollip recognize zTollip protein in Western blotting (WB) of lysates from fish embryos 5 days post-fertilization (dpf). β-catenin (detected with a human antibody) is present in embryos of this developmental stage. Antibody against human α-tubulin was used as a loading control. (C) Overproduction of zTollip inhibits the activity of Super8xTOPFlash luciferase reporter in Wnt3a-stimulated HEK293 cells. Ctrl, cells transfected with an empty pcDNA plasmid instead of a *ztollip*-encoding construct. Values are expressed as fold of untreated control, i.e. cells incubated with control-conditioned medium (CM) and transfected with an empty plasmid and the Super8xTOPFlash reporter. Data are mean ± SEM from 3 independent experiments; *P≤0.05 (Mann-Whitney U test).

**Fig 7:**
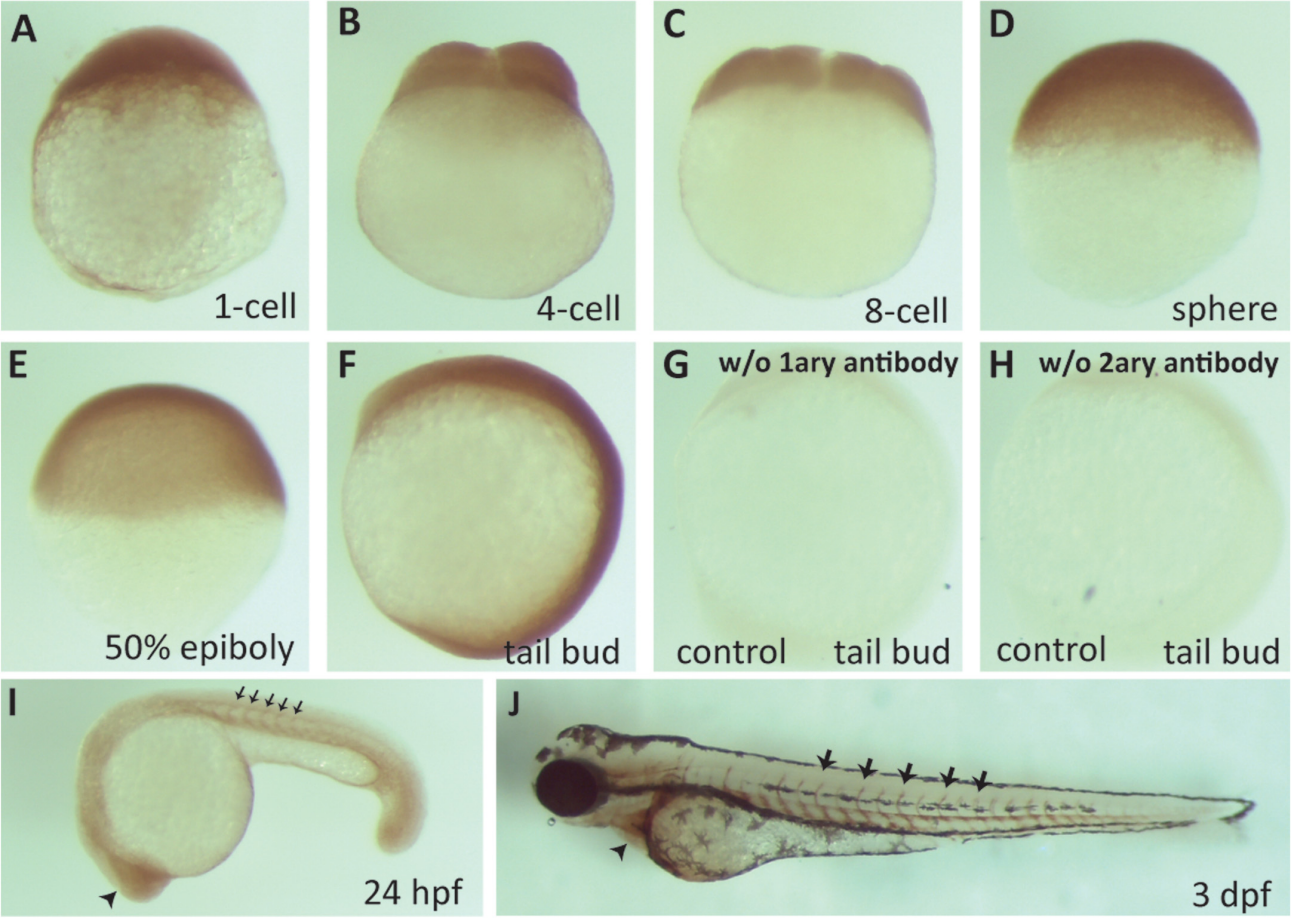
zTollip is broadly expressed during early zebrafish development. Immunocytochemistry analysis of zTollip protein localization in early embryonic development of zebrafish. Embryos fixed at various stages (indicated below the images) were incubated with primary antibodies against human Tollip, followed by secondary antibodies and chromogenic peroxidase-based detection. As controls for staining specificity, embryos at tail bud stage were processed omitting the primary antibody (G) or secondary antibody (H). zTollip expression is uniform from 1-cell stage until tail bud (A-F), but its expression increases in the head (arrowhead in I) and intersomitic regions (black arrows in I) at 24 hpf. At 3 dpf, zTollip is maintained in the intersomitic regions (arrows in J), but it is also expressed in the tissue surrounding the heart (arrowhead in J). Lateral views (A-H). Anterior to the left (I, J).

The genome of *Danio rerio* harbors a Tollip ortholog (GenBank accession number NM_207061.1) which encodes a well-conserved protein of 276 amino acids sharing the same domain structure [13] and 82% overall amino acid identity with its human counterpart (Fig 6A). Indeed, antibody against human Tollip recognized a band of an appropriate size in western blots from fish embryo lysates (Fig 6B). Immunohistochemistry staining during early development showed that zebrafish Tollip (zTollip) was maternally expressed and distributed throughout the blastoderm during the blastula stage (Fig 7A–7D). zTollip expression was also uniform until the end of gastrulation and beginning of somitogenesis (Fig 7E and 7F). During somitogenesis, particularly high expression was observed in the developing brain, eyes, and in the intersomitic regions containing blood vessels (Fig 7I). At 3 days post-fertilization (3 dpf), zTollip was also present around the heart (Fig 7J).

**Fig 8:**
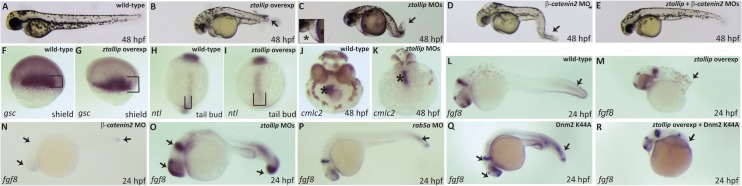
zTollip contributes to the canonical Wnt pathway in embryonic development. (A-E) Embryos overexpressing *ztollip* (B) or injected with either *ztollip* morpholinos (MOs) (C), or *β-catenin2* MO (D), or both (E) were analyzed for their morphological phenotype at 48 hpf. Typical phenotypes are shown. Lateral views with head to the left. Injection of *ztollip* mRNA led to reduction of posterior structures (arrow in B) compared with wild-type, control embryos (A). Knockdown of *ztollip* resulted in tail curvature (arrow in C) and pericardial oedema (asterisk in representative embryo in inset of C). Injection of *β-catenin2* MO caused morphological defects similar to those found in embryos overexpressing *ztollip* (compare B and D). Tail curvature and pericardial oedema in embryos overexpressing *ztollip* could be rescued by injection of 2 ng of *β-catenin2* MO (compare B and E). (F-R) *In situ* hybridization using different mRNA probes. Embryos injected with *ztollip* mRNA showed expanded expression of both the dorsal mesendodermal marker *goosecoid* (*gsc*) at 6 hpf (shield stage) (brackets in G) and the dorsal marker *notail* (*ntl*) at tail bud stage (brackets in I) compared to uninjected embryos (F and H, respectively). Expression of the cardiac marker *cmlc2* (J, K) indicated the failure of the heart to form the loop in *ztollip* morphants (asterisk in J) compared to wild-type embryos (asterisk in K) at 48 hpf. Expression of *fgf8* in the tail was reduced or absent in embryos overexpressing *ztollip* (arrow in M), while embryos injected with *ztollip* MOs showed an increase in *fgf8* expression in the brain and in the tail (arrows in O) compared to uninjected, control embryos (L) at 24 hpf. Embryos injected with 8 ng *β-catenin2* MO exhibited reduced *fgf8* expression (N). Blocking endocytosis by injecting embryos with 4 ng *rab5a* MO or by expressing the Dnm2-K44A displayed no clear effect on *fgf8* expression (arrows in P and Q). *ztollip* overexpression together with the Dnm2-K44A mutant showed a Tollip-like phenotype on the expression of *fgf8* (arrow in R).

To investigate the role of zTollip during development and its possible contribution to Wnt signaling, we injected synthetic *ztollip* mRNA in wild-type embryos. Upon injection of 200 pg of *ztollip* mRNA into 1-cell stage embryos (48 hours post-fertilization, hpf), embryos displayed a reduction of the posterior body, a strong reduction (even lack) of somites, and partial fusion of the eyes (Fig 8A and 8B).

Developmental defects caused by overexpression of *ztollip* mRNA were further analyzed by whole-mount *in situ* hybridization with mesendodermal, dorsal and neural markers. In *ztollip* mRNA-injected embryos, expression domains of mesendodermal markers such as *goosecoid* (*gsc*) appeared slightly enhanced compared to control embryos (Fig 8F and 8G). Moreover, the notochord was also slightly wider as visualized by the expression of *notail* (*ntl*) (Fig 8H and 8I). However, the expression of *fgf8* in the tail was reduced or even absent (Fig 8L and 8M). These phenotypes are reminiscent of those observed in zebrafish embryos where inhibitors of the canonical Wnt signaling such as Dkk1 were overexpressed [70] or when the canonical Wnt signaling was inhibited by *wnt8*/*wnt3* morpholino injection [71]. Indeed, downregulation of β-catenin2 by morpholino injection resulted in a decrease in *fgf8* expression (Fig 8N) and in defects similar to those observed in *ztollip*-overexpressing embryos (S4 Fig), indicating that zTollip could also function as an inhibitor of Wnt signaling during vertebrate development. Supporting this idea, overexpression of *ztollip* in HEK293 cells resulted in a reduction in the Super8xTOPFlash reporter activity (Fig 6C), as observed in the case of the human protein (Fig 1C). These data point to the conservation of Tollip function during vertebrate evolution.

Next, we knocked down *ztollip* by using various non-overlapping, independent morpholinos (MOs). We capitalized on the existence of two zebrafish mRNA variants, which differ by their 5’ UTR and ATG regions (http://zfin.org/ZDB-GENE-030131-8820, ZFIN IDs: ZDB-TSCRIPT-110325-2304 for a longer transcript and ZDB-TSCRIPT-110325-1396 for a shorter transcript). To block both mRNA variants, a combination of two morpholinos targeting each of them was used. Activation of Wnt signaling leads to pericardial oedema, smaller eyes, body curvature and failure of the heart to loop [72]. Similarly, morpholino-mediated downregulation of *ztollip* resulted in twisted tail, pericardial oedema and smaller eyes (Fig 8A and 8C). Moreover, *cmlc2* expression in the heart revealed that *ztollip* morpholino-injected embryos also failed to loop the heart (Fig 8J and 8K). In addition, and consistent with a role of Tollip in inhibiting Wnt signaling, *ztollip* morpholino-injected embryos showed an increase in *fgf8* expression in both the tail and the brain (Fig 8L and 8O). Most importantly, the defects displayed by embryos with *ztollip* knockdown (Fig 8C) could be rescued by downregulation of *β-catenin2* expression (Fig 8D and 8E).

To address whether the function of zTollip during zebrafish development is independent of its role in endocytosis, we overexpressed *ztollip*, while we simultaneously blocked the endocytic cycle in embryos by overexpressing the dominant-negative dynamin2 K44A mutant or downregulating the expression of *rab5a*, one of the key regulators of early endocytic steps [73, 74]. While *fgf8* expression was decreased upon overexpression of *ztollip* (Fig 8M), it was unaffected by injection of dynamin-K44A or *rab5a*-targeting morpholino (Fig 8P and 8Q), suggesting that endocytosis does not play a significant role in regulating expression of *fgf8*. Moreover, inhibiting the endocytic pathway by injection of dynamin-K44A did not rescue the negative effect of *ztollip* overexpression on the expression of *fgf8* (Fig 8R), further pointing to a role of zTollip in Wnt signaling independent of its endocytic activity.

## Discussion

In summary, our results identify a novel inhibitory activity of Tollip towards canonical Wnt signaling in mammalian cells and in zebrafish embryonic development. In addition to the universal core components of Wnt signaling, an increasing number of its regulators are being identified which may act in a more tissue-restricted or specialized manner [75]. Based on our data, we propose such a modulatory role for Tollip. At the molecular level this function is unrelated to endocytosis, but it is possible that Tollip acts by linking Wnt signaling with other signal transduction pathways.

The spectrum of pathways reported to be regulated by Tollip is broad, including innate immunity and NF-κB signaling [13, 15], TGF-β signaling [14], Rac1-dependent bacterial entry [16], sumoylation of nuclear and cytoplasmic proteins [18] and autophagic clearance of cytotoxic protein aggregates [17]. It is conceivable that the plethora of functions of Tollip could be linked via its role in autophagy and ability to bind ubiquitin (the latter being important also for the regulation of Wnt signaling, as we show here). On the one side, autophagy regulates various aspects of innate immunity, inflammation and pathogen elimination [76]. On the other side, selective autophagic degradation of cytoplasmic proteins affects a number of pathways [77], including Wnt signaling where autophagy-mediated elimination of Disheveled 2 [78] or of β-catenin [79, 80] were reported.

We propose that Tollip can inhibit the Wnt cascade at two stages. Upon ligand stimulation, Tollip reduces the pool of active β-catenin, possibly due to its reported role in the activation of GSK3β [62]. Additionally, under conditions of ligand-independent Wnt constitutive signaling (e.g. as occurring in cancer cells), Tollip inhibits the pathway downstream of β-catenin stabilization. Tollip was reported to interact with the components of sumoylation machinery and to regulate nucleocytoplasmic shuttling of proteins [18]. This could suggest an involvement of Tollip in sumoylation and/or regulation of nucleocytoplasmic distribution of β-catenin-associated proteins in the nucleus. Modification by sumoylation has been reported for TCF4 and LEF1 transcription factors [81, 82], as well as for their nuclear co-regulators such as the TBL1-TBLR1 co-repressor [83], Groucho [84], reptin [85], the CoCoA co-activator [86] or duplin/Chd8 [87], which in all cases modulates the output of Wnt signaling. In general, the involvement of Tollip at two steps of the Wnt cascade has already precedence in the NF-κB pathway, where Tollip functions at multiple stages and intracellular locations (plasma membrane, early and late endosomes) [8, 13, 15].

The Tollip ortholog in zebrafish has not been previously investigated. We show that zTollip is broadly expressed in early embryonic stages, and interfering with its function induces developmental phenotypes consistent with its proposed role in modulation of Wnt signaling. Thus, this function of Tollip is evolutionarily conserved between fish and mammals. Its physiological importance is further underscored by the fact that reduced expression of Tollip was reported in colon cancers [63]. It is therefore possible that the inhibitory activity of Tollip on canonical Wnt signaling could contribute to oncogenic processes in Wnt-dependent human malignancies.

## Supporting Information

S1 Fig esiRNA screen of selected endocytic proteins for the regulation of Wnt3a-dependent transcription. HEK293 cells were transfected for 72 h with 33 nM esiRNA targeting 80 indicated genes encoding soluble proteins regulating endocytosis. Cells were stimulated with Wnt3a-conditioned medium for 18 h before lysis and Super8xTOPFlash luciferase reporter assays were performed. Depletion of an established pathway activator β-catenin (*CTNNB1*) served as a positive control. esiRNAs targeting EGFP or 3 different regions of β-galactosidase served as negative controls. The pathway activity is presented relatively to the average of four non-targeting controls, which is normalized to one arbitrary unit. Average of non-targeting controls in cells without Wnt3a treatment (Ctrl NS) is shown to demonstrate the extent of stimulation. Data represent average of 3 independent experiments, error bars are SEM. Arrow indicates the results for Tollip knockdown.
(TIF)

S2 Fig (A) The level of Tollip protein is not changed upon overexpression of a dominant-negative K44A mutant of dynamin2 (Dnm2 K44A). Lysates from HEK293 cells transfected either with an empty plasmid (pcDNA) or HA-tagged Dnm2 K44A-expressing construct were blotted against Tollip and HA tag, with actin as a loading control. (B) Immunoprecipitation of overexpressed Tollip from lysates of HEK293 cells upon stimulation with Wnt3a or with control medium (CM) and proteasome inhibition by MG132. Western blots were probed for total ubiquitin and K48 or K63 ubiquitin subpopulations. Left panel; immunoprecipitates were probed with antibodies against Tollip, total ubiquitin (Ub), its K48-linked (Ub-K48) or K63-linked (Ub-K63) chains. Right panel; 10% of starting lysates taken for immunoprecipitation (input) were blotted against total ubiquitin (Ub) and Tollip, with GAPDH as a loading control.
(TIF)

S3 Fig Intracellular distribution of β-catenin upon overexpression of Tollip. HEK293 cells transfected for 24 h with myc-tagged Tollip: wild-type (wt), del.1 deletion mutant or M240A/F241A point mutant were stimulated with control medium (CM; A) or Wnt3a-conditioned medium (B) for 8 h. Cells were immunostained for β-catenin and myc tag (overexpressed Tollip), with DAPI indicating cell nuclei. Scale bar 20 μm.
(TIF)

S4 Fig Dose responses to the *β-catenin2* morpholino. Zebrafish embryos were injected at the 1-cell stage with *β-catenin2* morpholino (MO) at different concentrations (2 ng, 4 ng, 8 ng, 16 ng). Phenotypes of injected embryos at 48 hpf are shown, compared to an uninjected wild-type embryo. Lateral views with head to the left.
(TIF)
