# Supplementary figures and images for: Endocytic Adaptor Protein Tollip Inhibits Canonical Wnt Signaling
Source: PLoS One. 2015 Jun 25;10(6):e0130818. doi: 10.1371/journal.pone.0130818 (PMC4482507; doi:10.1371/journal.pone.0130818)

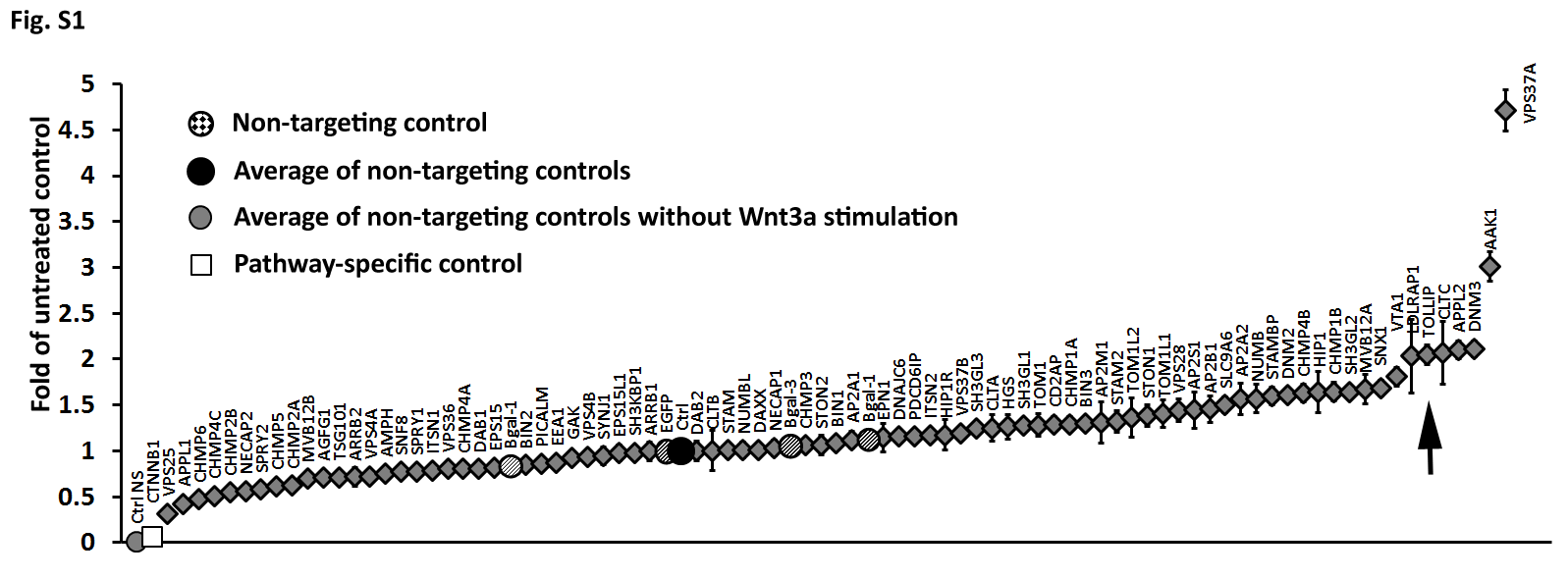

Supplement: S1 Fig — HEK293 cells were transfected for 72 h with 33 nM esiRNA targeting 80 indicated genes encoding soluble proteins regulating endocytosis. Cells were stimulated with Wnt3a-conditioned medium for 18 h before lysis and Super8xTOPFlash luciferase reporter assays were performed. Depletion of an established pathway activator β-catenin (CTNNB1) served as a positive control. esiRNAs targeting EGFP or 3 different regions of β-galactosidase served as negative controls. The pathway activity is presented relatively to the average of four non-targeting controls, which is normalized to one arbitrary unit. Average of non-targeting controls in cells without Wnt3a treatment (Ctrl NS) is shown to demonstrate the extent of stimulation. Data represent average of 3 independent experiments, error bars are SEM. Arrow indicates the results for Tollip knockdown. (TIF) [file pone.0130818.s001.tif]

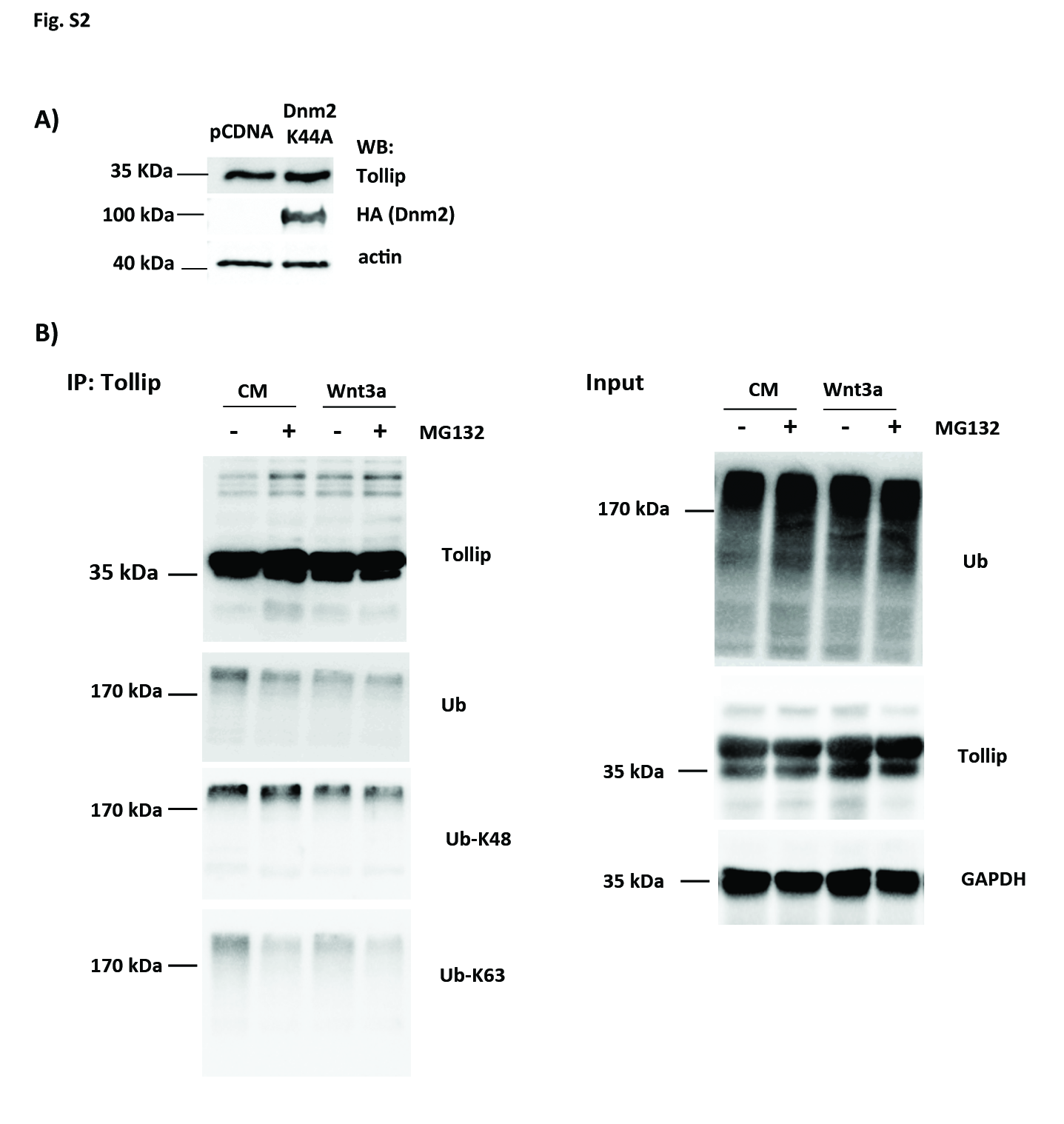

Supplement: S2 Fig — Lysates from HEK293 cells transfected either with an empty plasmid (pcDNA) or HA-tagged Dnm2 K44A-expressing construct were blotted against Tollip and HA tag, with actin as a loading control. (B) Immunoprecipitation of overexpressed Tollip from lysates of HEK293 cells upon stimulation with Wnt3a or with control medium (CM) and proteasome inhibition by MG132. Western blots were probed for total ubiquitin and K48 or K63 ubiquitin subpopulations. Left panel; immunoprecipitates were probed with antibodies against Tollip, total ubiquitin (Ub), its K48-linked (Ub-K48) or K63-linked (Ub-K63) chains. Right panel; 10% of starting lysates taken for immunoprecipitation (input) were blotted against total ubiquitin (Ub) and Tollip, with GAPDH as a loading control. (TIF) [file pone.0130818.s002.tif]

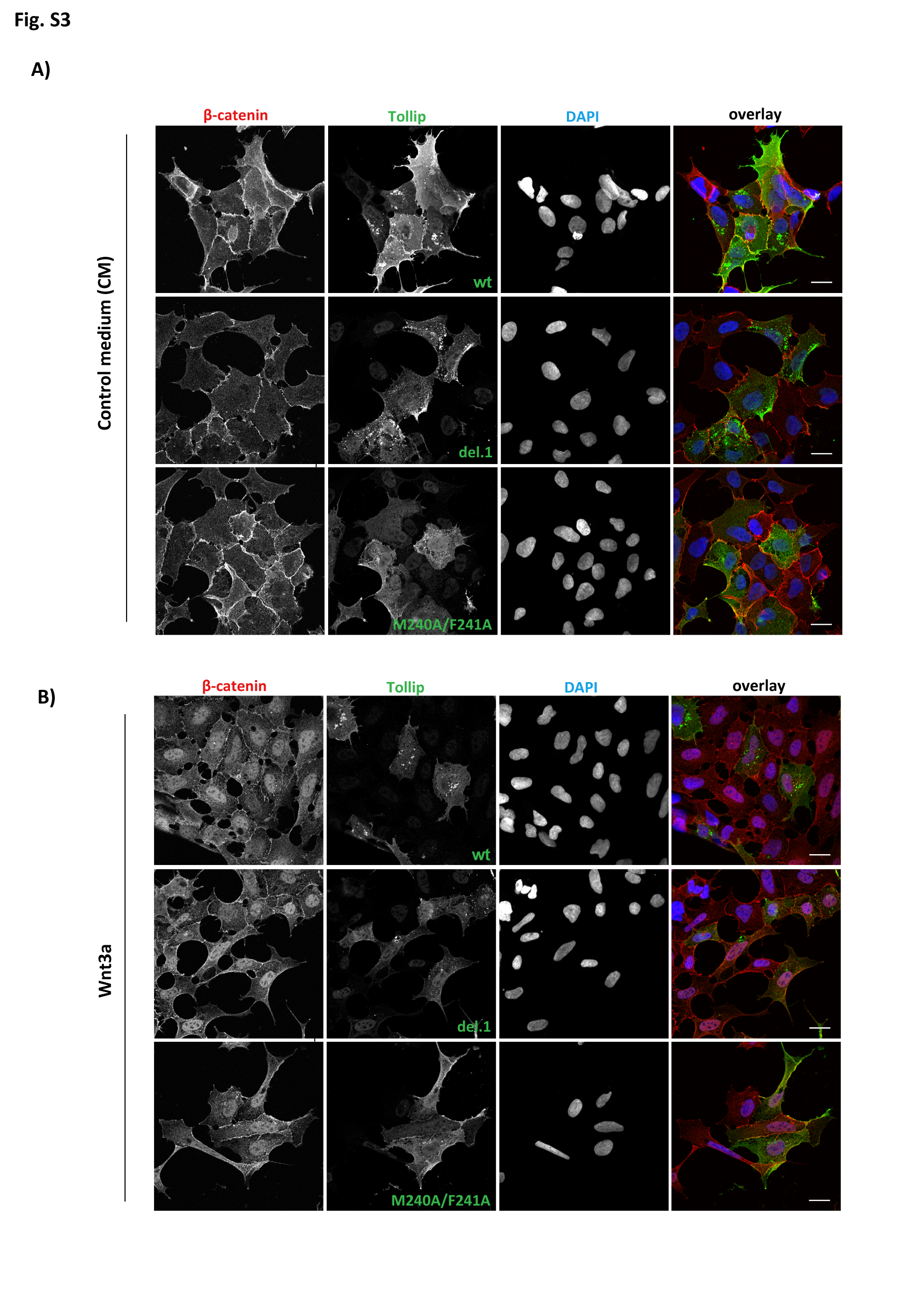

Supplement: S3 Fig — HEK293 cells transfected for 24 h with myc-tagged Tollip: wild-type (wt), del.1 deletion mutant or M240A/F241A point mutant were stimulated with control medium (CM; A) or Wnt3a-conditioned medium (B) for 8 h. Cells were immunostained for β-catenin and myc tag (overexpressed Tollip), with DAPI indicating cell nuclei. Scale bar 20 μm. (TIF) [file pone.0130818.s003.tif]

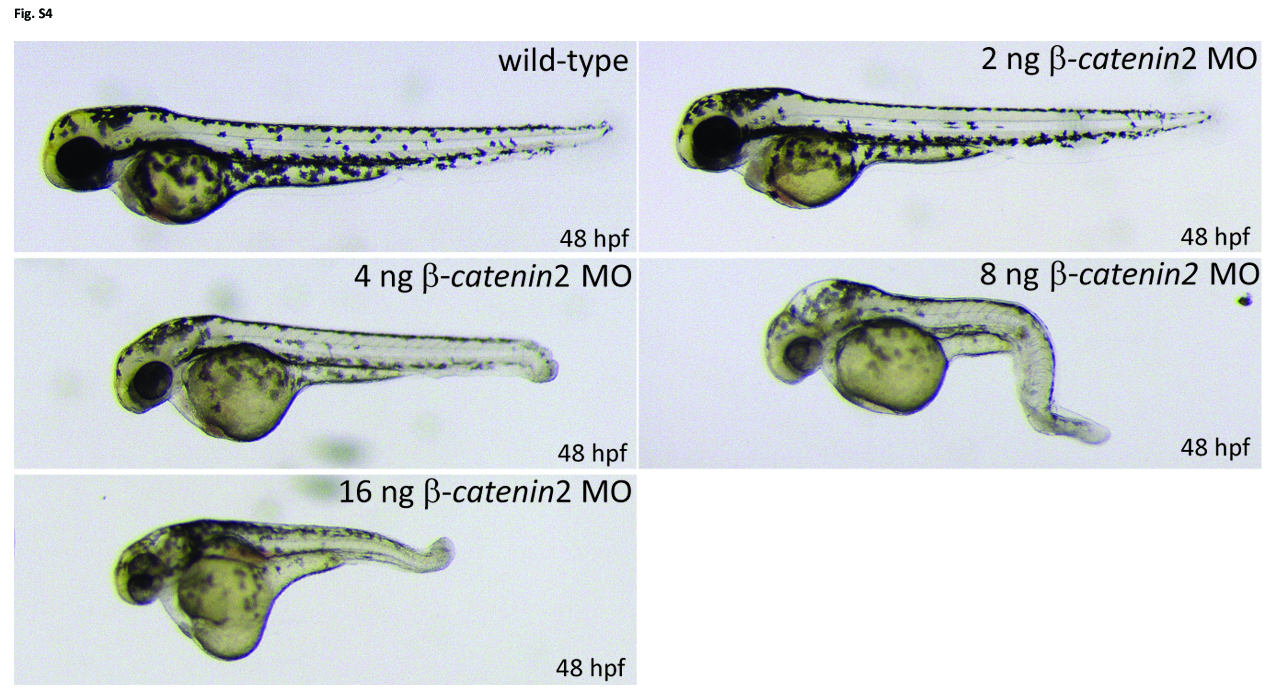

Supplement: S4 Fig — Zebrafish embryos were injected at the 1-cell stage with β-catenin2 morpholino (MO) at different concentrations (2 ng, 4 ng, 8 ng, 16 ng). Phenotypes of injected embryos at 48 hpf are shown, compared to an uninjected wild-type embryo. Lateral views with head to the left. (TIF) [file pone.0130818.s004.tif]
